# Determination of Optimal Flat-End Head Geometries for Pressure Vessels Based on Numerical and Experimental Approaches

**DOI:** 10.3390/ma14102520

**Published:** 2021-05-12

**Authors:** Paweł J. Romanowicz, Bogdan Szybiński

**Affiliations:** The Chair of Machine Design and Composite Structures, Cracow University of Technology, ul. Warszawska 24, 31-155 Cracow, Poland; bogdan.szybinski@pk.edu.pl

**Keywords:** flat ends, pressure vessels, stress relief grooves, numerical modeling, experimental tests, optimization, strain-gauge measurements, material characterization

## Abstract

The experimental and numerical analyses of the pressure vessels with different flat ends are presented and discussed in the paper. The main aim of the study is to propose the optimal flat head end geometry. The analyses are focused on the comparison of standardized geometries and with the proposed elliptical cut-out. The experimental tests with the application of strain-gauge measurements and numerical modeling of the pressure vessel are conducted. The behavior under low and high pressures and the influence of the residual welding stresses, material properties, and geometrical tolerances on the level of the plastic deformation in the flat end is discussed. It is presented that the rules given in the recent standard are not sufficient for optimal selection of the optimal geometry. It is observed that in certain geometries the deviations of the pipe thickness may lead to a significant increase of the equivalent stresses. The residual welding stresses have a significant influence on the stress and strain level—particularly in the stress relief groove (SRG). The performed study and comparison of the different geometries allow for the proposal of the optimal shape of the flat end. It appeared that the pressure vessels with SRG are the most optimal choice, particularly when elliptic shapes are in use. In some cases (i.e., pipe with wall-thickness equal to 40 mm and the flat end with circular SRG), the optimal configuration is reached for dimensions beyond the admissible by code range.

## 1. Introduction

Pressure vessels are widely used in various areas of industry, such as power or chemical engineering, and also as reactors, storage, railway or car cisterns as well as in non-industrial applications. The most common loads appearing in such structures are the inner or outer pressure and thermal loadings resulting from temperature differences between certain parts of the tanks. This results in the respective stress distributions, which influence in particular the pipe wall thickness. Other dimensions such as diameters are set according to the demands of the shape of the design [1]. The most crucial regions of the designing process are drain holes, inlets, stubs, manholes and other openings, and junctions between the pipe and the head, in all these regions high-stress concentration is observed, which influences the values of searched dimensions [2,3,4,5]. Due to the possibilities of catastrophic and unexpected failures, all pressure apparatuses are subjected to strict, internationally accepted regulations, which must be used in all stages of object operation, starting from the designing, through the manufacturing to the operation [5,6,7,8].

Depending on the type of application of the pressure vessel the different shapes of the vessel heads can be used [5]. The most often used pressure vessel heads are of curvilinear shapes such as elliptical, spherical, hemispherical, tori-conical, tori-spherical or conical and flat circular. The design of the whole structure should provide the required durability, strength and stability as well as it should be optimized with respect to the shape, size and vessel capacity [2,3,4,5,9,10,11,12,13]. The area of the greatest interest, due to the very high stresses appearance, are junctions between flat ends closures and pipes [14]. In the series of scientific papers [9,13,15,16,17,18,19,20,21] flat ends of pressure vessels have been presented as the possible alternative for the well-established curvilinear ends, which are predominant in common use [2,8,11,22,23]. The curvilinear shapes are much more frequently used in the case of vessels with bigger diameter values and rather thin-walled vessels. However, in the case of the use of cylindrical tubes with smaller than 400 mm diameters, dished ends are not commonly offered as standard products accessible on market. An even more complicated situation takes place when the vessels with the non-circular cross-sections are designed. Additionally, the procedure of spinning and pressing of the curvilinear tank heads demands the application of the cost generating molds or technologies, which becomes effective only in the case of mass production. When the tubes are subjected to relatively high pressures, the calculated wall thickness becomes rather big, and the use of flat ends seems to be also more rational and justified, due to their relatively simple form. Independently from the shape, all these ends are joined with the tubular parts of tanks through welding, which is a well-recognized and automatized technology nowadays [24,25,26].

The main drawback of all allowable in a view of codes forms of the flat ends used in practice results from the rapid, non-smooth change of shape of the inner edge of the vessel, which leads to the discontinuity of the curvature in this zone. This is the source of the stress concentration observed in the area of the change of the shape or its close vicinity [9,19,21]. The mentioned stress rise can be the source of the uncontrolled plasticization zone spread-out, crack nucleation and evolution and may result in premature failure of the structure consequently [27,28]. Such failure may take the form of an explosion, which must be definitely excluded from the exploitation practice. Therefore, to prevent such accidents different techniques for detection of the damages and ensuring the safety and operation efficiency described in Refs [29,30,31,32,33] can be applied.

**Figure 1:**
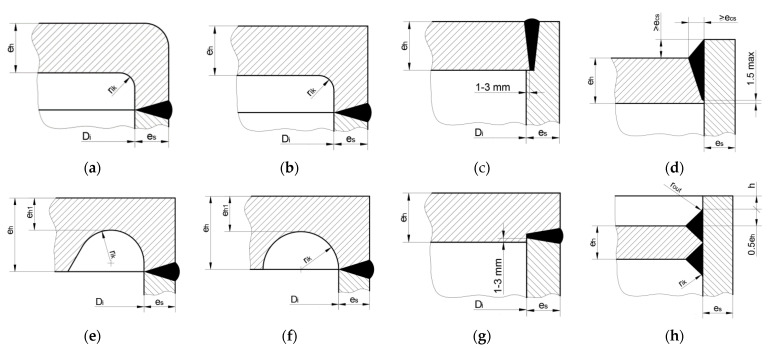
Standardized design of flat ends of pressure vessels (**a**) type “a” with fillet radius *r_ik_*; (**b**) type “b” with fillet radius *r_ik_*; (**c**) type “c”; (**d**) type “d”; (**e**) type “e” with SRG and with inclination; (**f**) type “f” with SRG; (**g**) type “g”; (**h**) type “h” with double-sided T-weld.

When using the same material for the tube and the flat end no longer the strength of the pipe is determining the maximum allowable operating pressure [16]. Because of no existence of analytical solutions for such structures (such as in the case of cylindrical vessels with ellipsoidal or hemispherical, or conical heads), the pressure value should be set in a series of numerical FE tests, which is the common practice in the designing. The detailed value of pressure reduction depends on the type of the applied flat end. This drawback can be partially minimized when using for flat end materials with much higher strength properties than for the tube. The detailed guidelines for the flat ends calculations are included in the respective designing codes such as EN 12952-3 [6] or EN 13445-3 [7], which suggest the admissible flat end shapes and give the respective mathematical formulas for defining their dimensions such as thickness and etc. The use of the second above given code is rather restricted and its limits were in detail described in the paper [9]. In this paper also certain shape modifications with the use of topology optimization are proposed to improve the strength properties of the studied structures. The first cited code gives much higher freedom in the choice of the shape of the flat end used. Here, the seven shapes of the flat ends are proposed and the respective formulas of calculations of their dimensions are given. Certain formulas are given in the forms of inequalities, which means that the particular dimensions can vary within the admissible limits and the optimal combination of dimensions providing minimum stress concentration can be searched. Such a detailed study was performed for the ends with the stress relief grooves (SRG), which at the first glance appear to be the most promising in a view of the possible application [15]. Because no profound and critical study of all the remaining admissible shapes for flat end solutions has been presented so far, the presented paper tries to fill this gap. The admissible forms of the flat ends by the EN 12952-3 code [6] are presented in Figure 1.

The main aim of the study is focused on the comparison of the standardized geometries of the flat ends and selection of the optimal shape of the flat end and experimental verification of the performed study. In the performed comparison, based on the finite element analyses, the standardized flat ends shapes are compared on the examples of thin- and thick-walled pressure vessels. The experimental and numerical studies have been carried for two different geometries of stress relief grooves of the commonly used flat end type “e”. These SRGs geometries are selected based on the numerical stress analyses—the optimal one (with the minimal value of the equivalent von Mises stress in the flat end) and the non-optimal another (with the maximal value of the equivalent von Mises stress in the flat end).

The paper consists of 5 sections and 4 Appendixes. The motivation and the description of the investigated problem as well as the literature review are given in Section 1. The principles of the design of the flat ends and description of the research program are described in Section 2. In this section, the numerical models there are also described. The results of the experimental tests of pressure vessels with the SRG and its comparison with the numerical analyses are presented in Section 3.1. The comparison of the behavior of the standardized flat ends is presented in Section 3.2. The summarizing discussion of the presented numerical and experimental study is given in Section 4. The concluding remarks are written in Section 5.

## 2. Materials and Methods

### 2.1. Vessels with Flat Ends

The first two presented above shapes of flat ends (Figure 1a,b) are the products of the machining or stamping. The shapes shown in Figure 1c,d,g are the simplest solutions from the manufacturing point of view—these only demands the preparation of flat slabs before final welding. However, the shape of the weld shown in Figure 1c,d,g should be treated only in the indicative form. This is due to the presence of the not-welded part of the tank and the flat head at the root of the weld. In this case, the very high-stress concentration is expected in the peak of the root of the weld after pressure imposing. In such a situation the recommendation is to use the complete penetration welds. The solution proposed in Figure 1e seems to be one of the most convenient from the manufacturing point of view and can be used in situations where no access to the inner part of the vessel is admitted. Additionally, the presence of the stress relief groove attenuates the stress concentration. Here, the code gives the series of inequalities, which determine the groove radius, its central location, and the minimum plate thickness in the groove [6,7,15,16]. Application of these formulas results in certain admissible ranges for the values for the radius *r_ik_* of the groove and location (*h*) of its center with respect to the bottom edge of the ending plate. In the so far performed numerical calculations, it was proven that the stress concentration strongly depends on the values of the groove radius. In the numerical tests, it was found that the position of the center groove must be set on an extension of the inner, bottom edge of the end plate [15], and the groove radius should be close to its maximum allowable value. The general rule for choice of the groove radius has not been given so far. Here, also the series of tests have proved that the application of the elliptical groove instead of the circular one (not admitted by the standards) significantly reduces the stress concentration, which confirms the common knowledge that non-circular notches are usually much more effective in notch analysis practice [34]. Additionally, the solution presented in Figure 1h is promising due to the presence of additional material around the top and the bottom welds, which helps to reduce the stress concentration. However, one must be aware that such a connection can be made only in cases where access to the inner part of the vessel is provided, which is not assured in arbitrary cases. This solution is also not fully determined with respect to the dimensions and opens certain areas of speculations on how to choose the radius *r_ik_* or what height *h* of the protruding part of the tank should be assumed. The common feature of all given above proposals is the formulae for the end plate thickness *e_h_* calculation, which is as follow:

(1) $$e_{h}=C_{1}\cdot C_{2}\cdot C_{3}\cdot d_{i}\cdot \sqrt{\frac{p_{c}}{f}}$$

**Figure 2:**
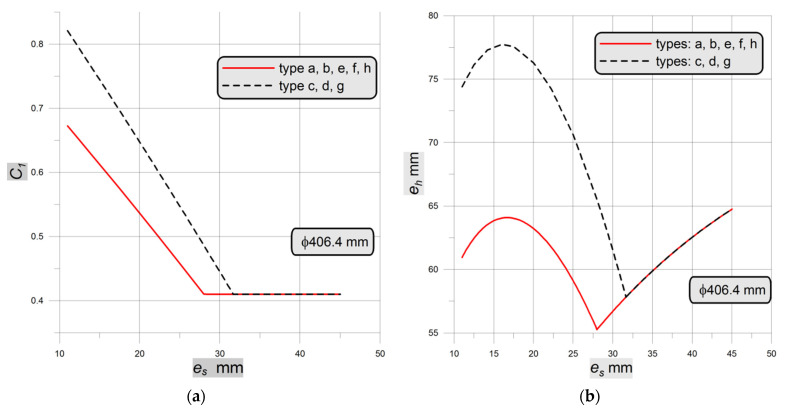
Dependency graph of: (**a**) *C*_1_ constant; (**b**) endplate thickness *e_h_*.

In the above equation, *d_i_* is the inner tank diameter, *p_c_* is the operating pressure and *f* stands for the admissible stress. Constants *C*_2_ and *C*_3_ are set to 1.0 when considering the tanks with the circular cross-sections and if there are no holes in calculated heads. The choice of the constant *C*_1_ is a little bit complex—it could be taken from the plots given in code [6] or directly calculated from the formulas:

(2) $$\begin{array}{l} C_{1}=(-1.05725-1.60840\cdot x+0.116245\cdot x^{2}-0.288657\cdot x^{3})\cdot \frac{p_{c}}{f}\\ +0.54+0.324245\cdot \frac{1}{x}-0.668534\cdot \frac{1}{x^{2}}+0.634377\cdot \frac{1}{x^{3}} \end{array}$$

for the butt welded end plates shown in Figure 1a,b,e,f,h or

(3) $$\begin{array}{l} C_{1}=(-1.13850-2.80682\cdot x+0.841379\cdot x^{2}-0.517434\cdot x^{3})\cdot \frac{p_{c}}{f}\\ +0.68+0.231694\cdot \frac{1}{x}-0.440285\cdot \frac{1}{x^{2}}+0.550637\cdot \frac{1}{x^{3}} \end{array}$$

for the ends joined through the T-weld, namely these given in Figure 1c,d,g. In both cases, *x* is the ratio between the real wall thickness assumed for the tank to the vessel wall thickness calculated from the common strength formulae. Additionally, the condition says that if the calculated from (2) or (3) equation constant *C*_1_ is lower than 0.41, then its value should be set to 0.41. In the presented study it is assumed that the real wall thickness is equal to the calculated one, which means that *x* = 1.0. For the pipes with Ø406.4 mm and thicknesses varying from 11.0 mm to 45.0 mm (standard production program) the distribution of the constant *C*_1_ and the thicknesses of the end plate *e_h_* for types “a” to “h” are shown in Figure 2.

The dependency shown in above Figure 2 is valid for the case when the material of the tank and the head are the same. As it can be observed in Figure 2b, the thicknesses of the endplates for all constructional cases do not constantly increase with the rise of the tank wall thickness. The proportional increase of the endplate thickness—which seems to be logical—is observed from the moment when the *C*_1_ value becomes constant and equal to 0.41.

### 2.2. Experimental Tests of Pressure Vessels

**Figure 3:**
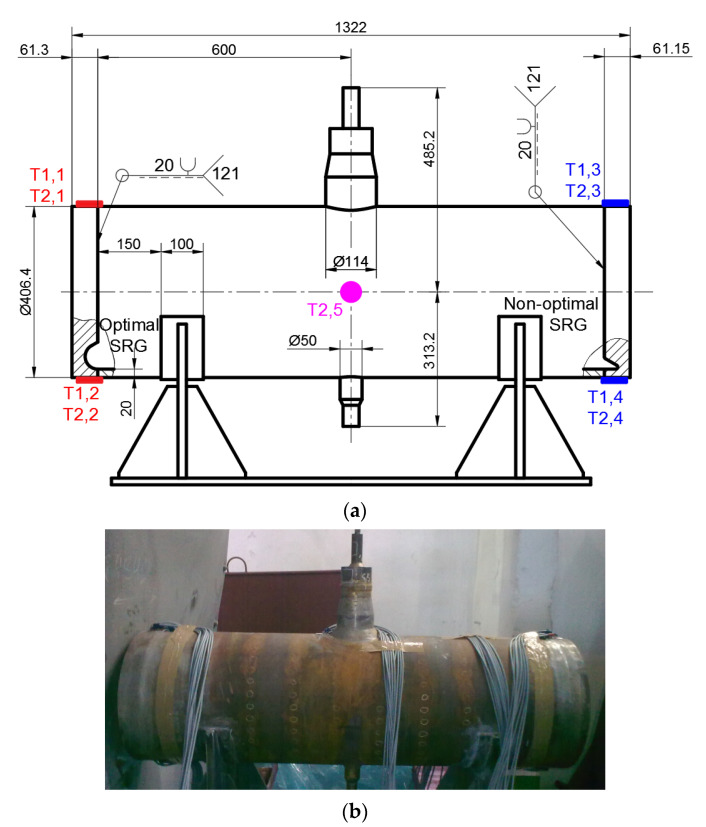
Tested pressure vessel: (**a**) geometry of tested pressure vessel with two different flat ends with marked location of the strain gauges sets (dimensions in mm); (**b**) tested vessel with additional equipment.

The experimental and numerical studies were conducted for pressure vessels (Figure 3) made of chrome-molybdenum 16Mo3 steel alloy. Such steel is used in the manufacturing of pressure vessels and industrial boilers working at elevated temperatures. The numerical analyses are verified by the experimental tests of pressure vessel with the use of strain gauge measurements. For such purposes, the vessel with two different shapes (optimal and non-optimal with respect to the minimal plastic strains [17]) of flat ends type “e” were manufactured and tested. Such validation of FEM analyses required accurate modeling of the σ-ε curve of flat end material. For such purposes, detailed experimental tests for the applied material were conducted. The detailed information about the material (chemical composition), performed experimental tests for 16Mo3 steel alloy, and their results (mechanical properties, material behavior) are reported in Appendix A.

The external nominal diameter of the vessel was equal to Ø406.4 mm, and the nominal thickness of the cylindrical tube was 20 mm. The detailed measurements of the real tube thickness and diameter were made and presented in Appendix B. The length of the tube equal to 1200 mm was adopted to avoid the influence of the boundary effects and additional equipment (such as connector, supports and pressure gauge) on the measured strains in both flat ends.

**Figure 4:**
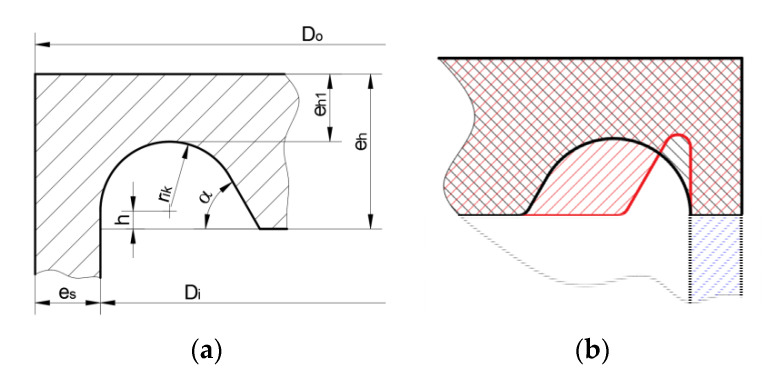
Flat end with stress relief groove: (**a**) geometry and dimensions; (**b**) comparison of tested flat ends with optimal and non-optimal stress relief grooves.

The experimental tests were made for flat ends designed concerning the Standards [6] but with different geometries of the stress relief grooves. The dimensions of the flat ends are presented in Figure 4a. In the previous studies [17] it was presented that the dimensions of the stress relief groove may vary within a wide range. It results in significant differences in the stress relief groove shape. Moreover, this leads to the different strengths of the structure [15]. It was observed that in the area of the admissible dimensions of the flat end geometry (determined by the Standards [6]) there are available solutions, for which there are significant differences in the equivalent von Mises stresses and plastic strains [9]. Due to this fact, the experimental tests were performed for two solutions (from the admissible area specified by the Standard [6]) in which the smallest (geometry no.1 optimal) and the highest (geometry no.2 non-optimal) plastic strains occur.

The maximal admissible operating pressure was calculated with respect to the Standard [6] and for the minimal Yield limit of the pipe (308 MPa) and was equal to 23.5 MPa. In the presented study the operating pressure was set at 22 MPa. For such parameters, the pressure of the hydraulic waterproof test was equal to 35 MPa. The geometries of both stress relief grooves were determined based on the measurements of the outer diameter and thickness of the cylindrical pipe and assumed operating pressure. The optimization process was performed for the mean values of the real diameter and thickness. The geometries of these two solutions are compared with each other in Figure 4b and dimensions are summarized in Table 1. It can be seen that these solutions mainly differ in fillet radii of the stress relief grooves. Such radius has the greatest impact on the stress concentrations and reduction of its leads to a strong increase in stress and strains. More details about the optimization of such stress relief grooves are given in References [15,17].

**Figure 5:**
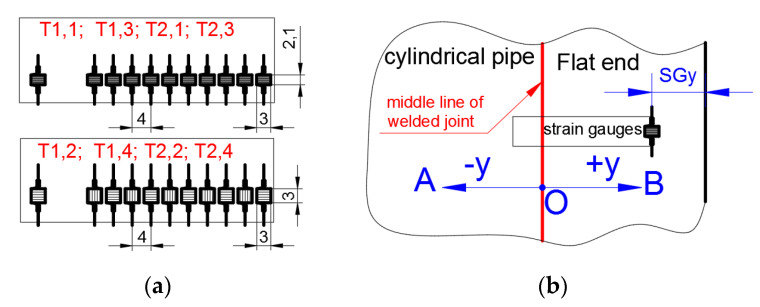
Information about strain gauges: (**a**) dimensions of strain gauge chains; (**b**) location of strain gauges on vessel surface (length *SGy*) and definition of axis *y* and points (A, O, B) for presentation of results.

The verification of the numerical analyses of the stress concentrations in flat ends is made by comparison of the strains measured on the external surface of the vessel with the results of the numerical FE analyses. The experimental measurements of strain were made with the use of strain gauge chains. Such chains have been marked as T*j*,*k*, where *j* = 1,2 means the measurement series and *k* = 1,2,…5 means location of the strain gauge chain (Figure 3). There were used sets with ten measuring grids—parallel to the chain axis KY11-4/120 (sets T1,1, T1,3, T2,1 and T2,3) and alternating five parallels and five perpendiculars to the chain axis KY41-4/120 (sets T1,2, T1,4, T2,2 and T2,4). Each chain has one compensating strain gauge. The detailed information about the dimensions of the strain gauge chains is given in Figure 5a. In the 2nd series of measurements, strain gauge rosette RY7x-10/120 for additional controlling the internal pressure (set T2,5 in Figure 3) was used.

The location of the strain gauge sets was determined by the use of the FE analyses in ANSYS software [35]. There were determined locations (Figure 3) in which the highest strains on the external surface may occur. The locations of the strain gauges from the end of the flat ends are determined by the length *SGy* according to Figure 5b and is given in Table 2.

**Figure 6:**
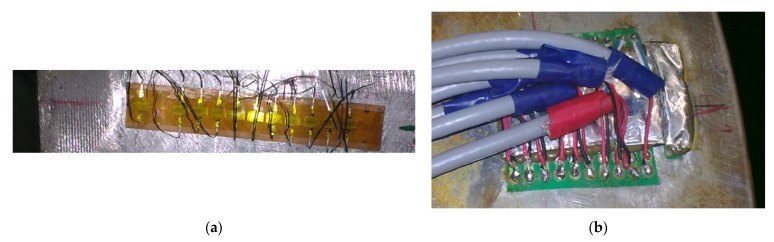
Photographs of strain gauges chain glued on tested pressure vessel: (**a**) strain gauge before connecting the wires; (**b**) finished connection.

Glued strain gauge chain on the external surface of the vessel (end of the flat end is visible at the left-hand-side) before connecting the wires is shown in Figure 6a. The finished connection with made and secured connecting wires is presented in Figure 6b.

The experimental tests were performed in two series with the same loading cycles. The first series of measurements (namely 1.*i*; where “*i*” is a cycle number) were made immediately after manufacturing the vessel. In this case annealing of the vessel was not performed to investigate the influence of the residual stresses in welded joints on the behavior of the stress relief grooves in flat ends. The second series of measurements (namely 2.*i*; where “*i*” is again a cycle number) were made after annealing of the vessel structure.

**Figure 7:**
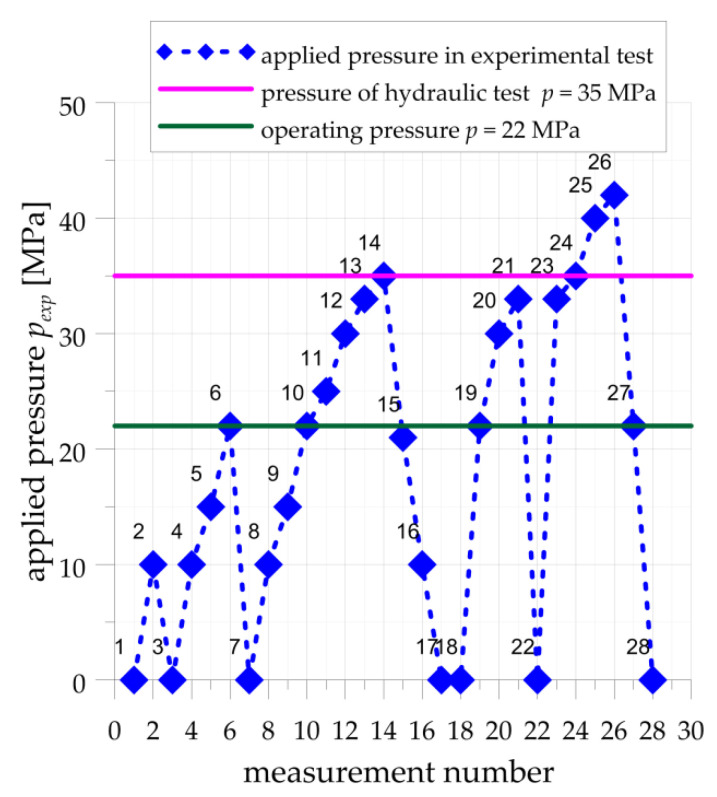
Applied load cycles with nominal values of internal pressure and number of measurement points.

During the experimental tests, the structure was loaded by the internal pressure with the different values in the range *p_int_* = 0–41 MPa. The measurements of strains were made for 28 cycles with various values of the internal pressure. The detailed scheme of applied load cycles is given in Figure 7. The experimental testing procedure has consisted of the different 5 loading-unloading cycles. The first cycle (measurements 1–3) was a trial test made for checking the measurement system. The maximal applied pressure in this cycle was equal to 10 MPa. For such loading, there should be no plastic strains in the structure, including the areas with stress concentrations around the stress relief grooves. In the second cycle (measurements 3–7), the maximal applied pressure was equal to the operating pressure *p_c_* = 22 MPa. The first plastic deformations, based on the FEM analyses, were expected for *p_int_* = 15 MPa. In the third cycle (7–17), the measurements were made for previously tested pressures (verification of the repeatability of the selected results) and higher pressure till the pressure of the hydraulic proof test *p_int_* = 35 MPa. In the fourth cycle (18–22), the maximal pressure was equal to 33 MPa, and the main aims of this load cycle were to compare structure response with previous load cycles. The measurements in the last fifth load cycle (22–28) were made for pressures (*p_int_* = 40 MPa and *p_int_* = 41 MPa) higher than the pressure of the hydraulic test until the full plasticization of the weakest cross-sections of the pressure vessel occurred.

In the 1st measurement series, cycles 18–28 were realized after the six-week break. It should be noted that a new zero state for strain gauges was set in measurement no. 18. Because of this, the strain state measured in cycle no.17 was summed with the results obtained in cycles 18–28 in the 1st series. All measurements in the 2nd series were made continuously at one day.

### 2.3. Numerical Analyses of Flat Ends

**Figure 8:**
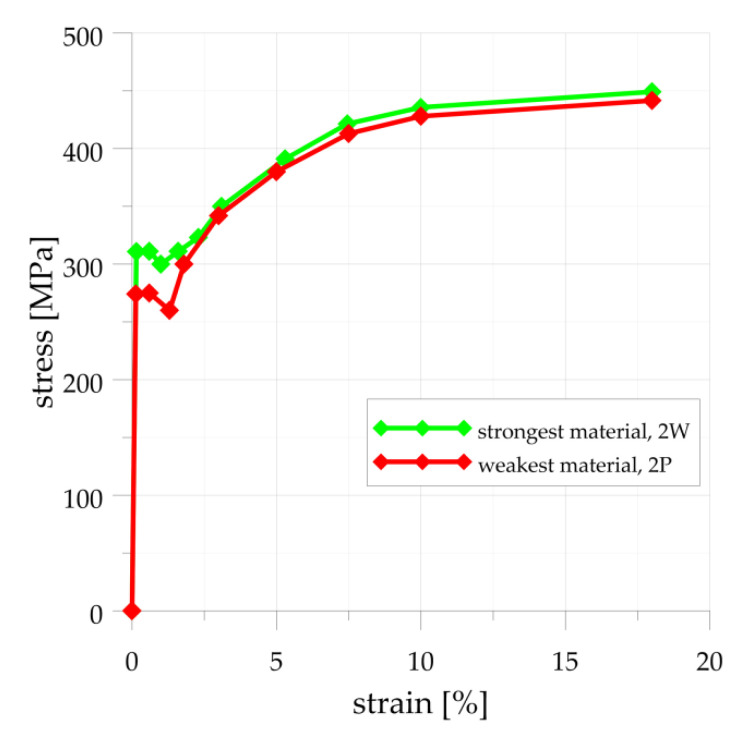
Stress–strain curves implemented for numerical analyses in Section 3.1.

**Figure A1:**
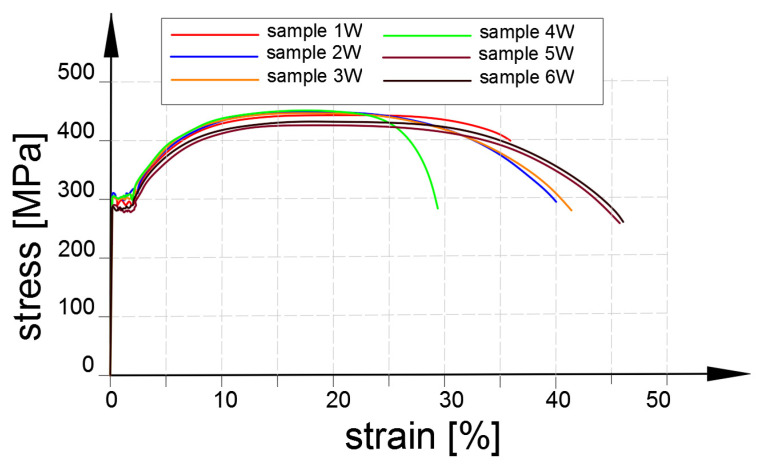
Engineering stress—strain curves for 16Mo3 + N alloy steel. Samples were cut from flat end in longitudinal direction (set 1a).

**Figure A2:**
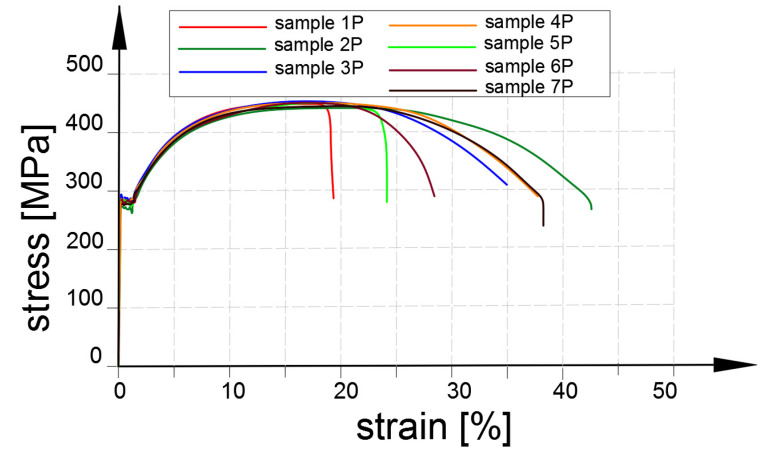
Engineering stress–strain curves for 16Mo3 + N alloy steel. Samples were cut from flat end in transversal direction (set 1b).

The FE analyses are made to compare the numerical results with the experimental ones obtained both for optimal and non-optimal configuration of the SRG cut in the endplate of type “e” (Figure 1). The second part of numerical calculations is made to assess the effectiveness of various types of flat ends recommended by code [6]. These two sets of simulations use different material models in numerical analysis. In the performed experimental tests the applied internal pressure was high enough to cause the rise and development of the plastic deformations in the vessel. That is the reason why the elastic-plastic material model is adopted for FE analysis. The data for this model follow the tension tests of 16Mo3 steel results (see Appendix A) and the material model with the multi-linear hardening for plastic strains is applied. Here, to get the boundary assessment of the numerical results the material model parameters are established for the “strongest” (2W material, Figure A1) and the “weakest” material (2P material, Figure A2) samples are set and applied in FE calculations. The characteristic stress–strain curve used for simulations in Section 3.1 is shown in Figure 8. The vertexes of that curves are determined by the respective results of tension tests.

**Figure 9:**
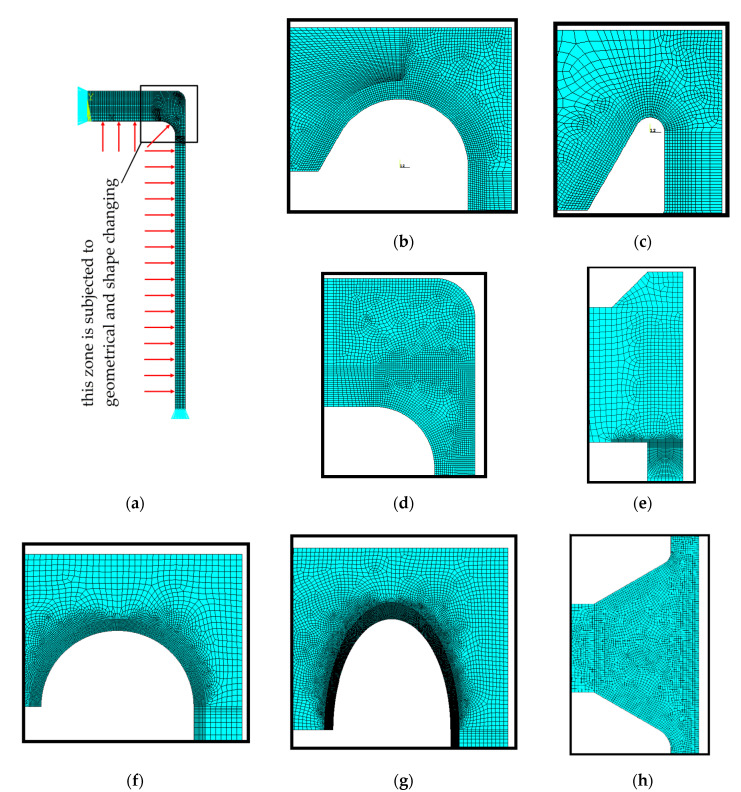
Numerical models for analyses of pressure vessels with flat heads: (**a**) finite element mesh with boundary conditions; (**b**) mesh for experimentally tested endplate type “e” with optimal SRG; (**c**) mesh for experimentally tested endplate type “e” with non-optimal SRG; (**d**) mesh for endplate type “a”; (**e**) mesh for endplate type “d”; (**f**) mesh for endplate type “f”; (**g**) mesh for proposed elliptical SRG; (**h**) mesh for endplate type “h”.

The size and dimensions of the investigated vessel are introduced into the FE model (Figure 9a). The parts of the finite element meshes used in analyses are shown in Figure 9b,c. It is also worth adding that the main aim of such study is a comparison with the results of the 2nd measurements series, in which the vessel is subjected to the annealing process before the tests. This enables to omit the problem of the residual welding stresses, which may appear after manufacturing and perturb the experimental results.

The second part of the analyses presented in Section 3.2 is mainly made for the nominal dimensions corresponding to the formerly investigated in experiment vessel (i.e., Ø406.4 × 20 mm and full-length 1200 mm, flat ends at both ends). Here, the numerical calculations are performed for various types of the recommended flat endplates (Figure 1a–h). The crucial parts of the respective FE meshes, covering the most strenuous junction between the pipe and the endplate, are shown in Figure 9d–h. In this case, all numerical calculations are made for a purely linear elastic material model and the maximum stresses observed in simulations have only informative meaning. For 16Mo3 steel typical strength properties are assumed, so that the admissible stress *k* for the cylindrical part of the vessel is set to 180 MPa. Using this value, the nominal internal pressure was set to *p_c_* = 18.6335 MPa. In order to evaluate the durability of the chosen flat endplate, the ratio λ between the maximum numerically calculated stress and the admissible value *k* is introduced. In such a sense the lowest value for λ means the most effective geometrical shape for the endplate.

For the detailed calculations, the ANSYS FE code [35] is used. Certain simplifications of the analyses are introduced, which rely on the use of the symmetry of the structure geometry and the symmetry of the loadings. Using these assumptions, the analyses can be reduced to the axis-symmetric problem. Such an approach allows for a substantial reduction of the size of the numerical task to solve, also the relatively dense meshes can be applied in the notch zones. In detailed FE calculations the plane finite element PLANE183 with axisymmetric option switched on is used. This element has a quadratic approximation of the displacements and is particularly suitable for problems with irregular meshes. In all performed calculations in this step, the quality of the solutions was controlled by the structural energy error, which was not greater than 2% in all studied cases.

## 3. Results

### 3.1. Experimental Tests of Pressure Vessel

The experimental tests performed in the research program included the detailed characterization of different parameters which can influence the behavior of the investigated pressure vessel. Such experimental studies contained the determination of the real material stress–strain curves (Appendix A), the real external diameter of the pipe (Appendix B) and the real thickness of the pipe (Appendix B). The experimental research program was also prepared in order to enable the verification of the accuracy of the obtained results. Because of this, the two different measurement systems were used for verification of the internal pressure. The main system for controlling of the pressure was applied on the hydraulic pump—these results are not attached in the paper, and verification of the pressure was made through the measurements of the strains on the external surface of the vessel (Appendix C). The verification of the accuracy of the strain-gauge measurements is presented in Appendix D.

#### 3.1.1. Numerical vs. Experiments—General Remarks

**Figure A3:**
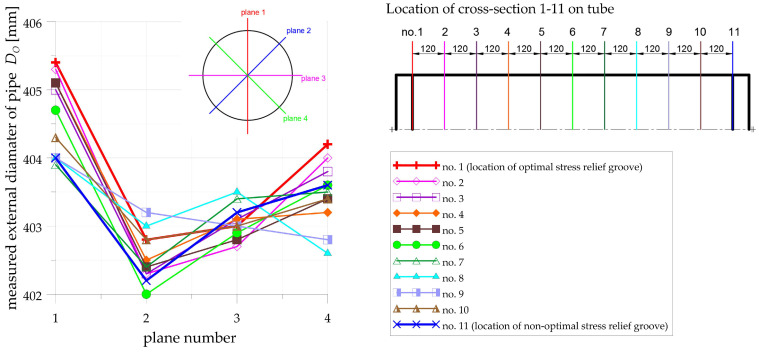
Measured diameters of cylindrical pipe of vessel in different cross-sections.

**Figure A4:**
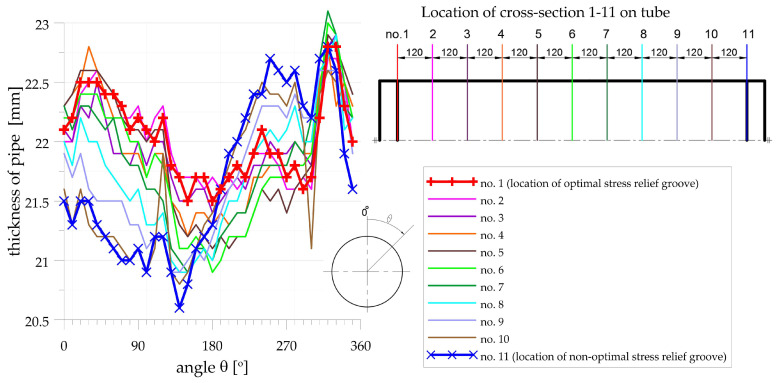
Measured thickness of cylindrical pipe of vessel in different cross-sections.

First of all, the influence of the welding process on structure behavior is studied by comparing the experimental results for both measurement series. The experimental results are also compared with the FEM solutions. Three sets of geometries (with different thicknesses, outside diameters and materials) are investigated in the numerical analyses. First of all, it must be noted, that the measured thickness and outer diameter of the cylindrical pipe significantly differ from the nominal ones, however, within the required and typical tolerance for pipes [36]. Because of this, in the first two cases, the mean real dimensions in particular cross-sections (more information can be found in Appendix B in Table 1 and Table A3 and Figure A3 and Figure A4) are assumed in the numerical models. In the case of the optimal SRG the mean thickness is 22.0 mm and the mean outer diameter Ø403.85 mm, and in the case of the non-optimal SRG the mean thickness is 21.7 mm and the mean outer diameter Ø 403.25 mm. In figures in Section 3.1.2 and Section 3.1.3, the results for the strongest material 2W are presented by the continuous green line, and the results for the weakest material 2P are presented by the continuous red line (σ−ε curves are given in Figure 9, Figure A1 and Figure A2). There are also shown results for the minimal measured geometrical parameters (the thickness for optimal SRG—21.5 mm, non-optimal SRG—20.6 mm—see Table A3 and Figure A3 and Figure A4) and the weakest material 2P by a brown continuous line.

#### 3.1.2. Numerical vs. Experiments—Optimal Stress Relief Groove

**Figure 10:**
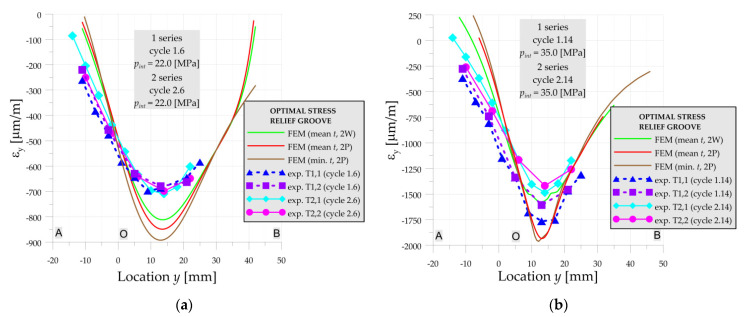
Numerical and experimental strains on line A-O-B, optimal SRG, 1st and 2nd series: (**a**) cycles 6; (**b**) cycle 14.

In the case of the optimal SRG there are no significant differences in strain distributions in measurements in both series for pressures *p_int_* = 10 MPa—cycles 4 and operating pressure *p_int_* = 22 MPa—cycles 6 (Figure 10a). The experimental strains are slightly smaller than numerical ones in both above loading cycles. Based on the numerical solutions given in Figure 10a, it is also observed, that the influence of the varying dimensions in the range of tolerance and the strength of the material is negligible for optimal SRG and operating pressure.

For higher pressures (*p_int_* = 35 MPa—Figure 10b) the influence of the residual strains in the 1st series (caused by welding process) appears and results in higher strains in the optimal SRG. In this case, the significant influence of the material strength on the strain distribution in the numerical solution can be also observed. However, there is still no visible influence of the geometry change on the strain distribution.

**Figure 11:**
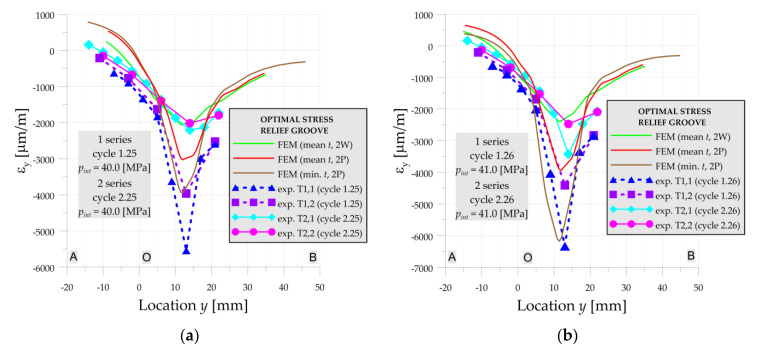
Numerical and experimental strains on line A-O-B, optimal SRG, 1st and 2nd series: (**a**) cycles 25; (**b**) cycle 26.

Further increase of the internal pressure causes more and more variations of the results. For *p_int_* = 40 MPa (Figure 11a) experimental strains for not-annealed vessel (series 1) are almost 2–3 times larger than after annealing (series 2) of the vessel. Similar behavior is observed when comparison of these experimental tests with the numerical solution performed for structure with mean dimensions is made. The maximal longitudinal strain is equal to 5544 μm/m. On the other hand, the strains measured after annealing in the 2nd series are in good agreement with the numerical solution. Such high differences in experimental results of 1st and 2nd measurement series are caused by the existence of the residual stresses in the 1st series due to the welding process and the relatively short distance between stress concentration and weld joint (*y* = 0—point O is the center of the weld joint).

In the case of the high loadings (Figure 11) the geometry also had a large influence on the structure behavior. The slight change of the thickness (within the range of tolerance of the pipe—reduction of the thickness from 22.0 to 21.5 mm) leads to a large increase of plastic deformation. This effect becomes more and more important with the increase of the pressure as can be seen in Figure 11b for *p_int_* = 41 MPa.

Finally, for the optimal SRG, it can be observed that the existence of the residual stresses caused by welding (strains) in 1st series has a comparable influence on the increase of the measured strains as a decrease of the thickness of the tube in the numerical solution.

**Figure 12:**
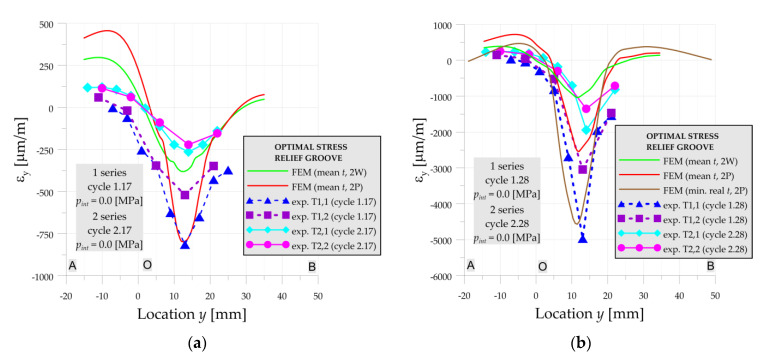
Numerical and experimental strains on line A-O-B, optimal SRG, 1st and 2nd series: (**a**) cycle 17; (**b**) cycle 28.

The influence of the material properties, thickness as well as residual welding stresses is also visible on the examples of residual strains presented in Figure 12 for cycles with numbers 17 and 28. Due to the close occurrence of the stress concentrations to the welding joint and heat-affected zone, the residual welding stresses significantly affect the load capacity of the investigated vessel with optimal SRG.

#### 3.1.3. Numerical vs. Experiments—Non-Optimal Stress Relief Groove

**Figure 13:**
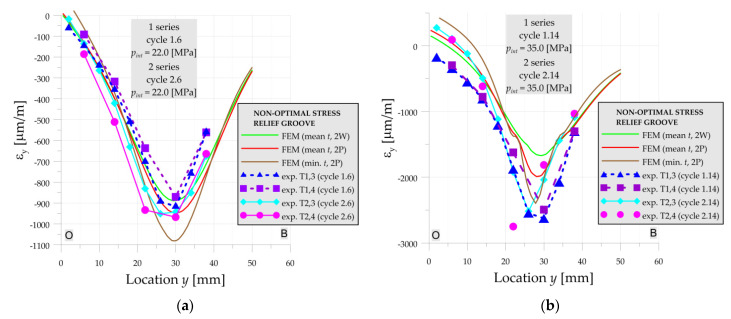
Numerical and experimental strains on line O-B, non-optimal SRG, 1st and 2nd series: (**a**) cycles 6; (**b**) cycle 14.

A similar comparison of the experimentally measured strains in both series and calculated in the numerical solutions are carried for non-optimal SRG. For the operating pressure *p_int_* = 22 MPa (cycle 6—Figure 13a) the experimentally measured strains in both series are in good agreement with the numerical solution.

**Figure 14:**
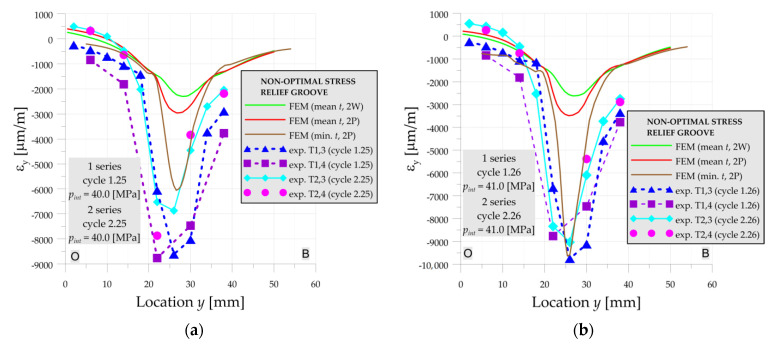
Numerical and experimental strains on line O-B, non-optimal SRG, 1st and 2nd series: (**a**) cycles 25; (**b**) cycle 26.

**Figure 15:**
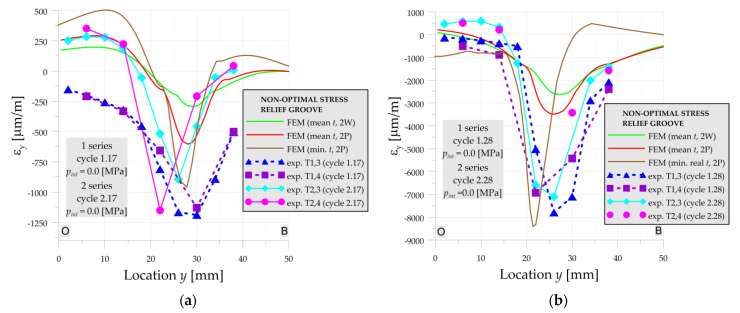
Numerical and experimental strains on line O-B, non-optimal SRG, 1st and 2nd series: (**a**) cycle17; (**b**) cycle 28.

Similar to the case of the optimal SRG, the differences in the material properties for operating pressure are negligible, however, there is a visible weakening of the structure by reducing the pipe thickness (mean thickness is 21.7 mm, and minimal 20.6 mm—Table A3). In contradiction to the optimal SRG, with the increase of the internal pressure, the difference between experimental and numerical (for mean dimensions) results has rapidly grown in non-optimal SRG. The obtained experimental strains for both series (including loading and unloading cycles) are in better agreement with the numerical solution for the case with the minimal measured thickness of the pipe (continuous brown line in Figure 13, Figure 14 and Figure 15).

Comparing the results for non-optimal SRG with the results for optimal SRG it can be seen that the influence of thickness reduction has a stronger impact on the non-optimal SRG. In the case of high pressures, the decrease of the pipe thickness from 21.7 to 20.6 mm increases of the maximal strains 2× and 3× times for 40 MPa (Figure 14a) and 41 MPa (Figure 14b), respectively. This may be caused by the larger reduction of the minimum thickness in the case of the non-optimal SRG and/or sharper notch caused by the smaller fillet radius (see Figure 4b). Moreover, higher magnitudes of strains are achieved in the non-optimal SRG. The highest differences are visible for the 2nd series after annealing of the vessel. For example, for the pressure 41 MPa, the highest strain in non-optimal SRG (Figure 14b) is 2.5× larger than in optimal SRG (Figure 11b).

The next difference in the behavior of the optimal and non-optimal SRG is that the experimental results in non-optimal SRG are similar in both measurement series. The lack of a clear influence of residual welding stresses (which has a strong influence on the strain level in the optimal SRG) can be explained by the larger distance of the weld position to the zone with stress concentration in the non-optimal SRG.

#### 3.1.4. Numerical Study of Plastic Strain Development

**Figure 16:**
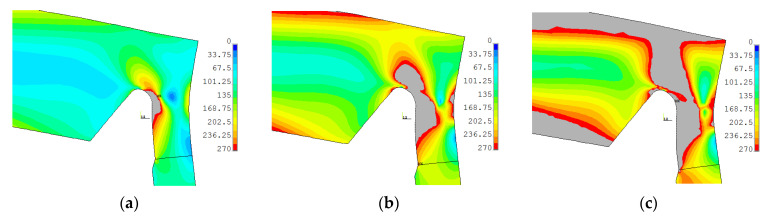
Equivalent von Mises stress in non-optimal SRG with mean dimensions, material 2P for: (**a**) cycle 6—22 MPa; (**b**) cycle 14—35 MPa; (**c**) cycle 26—41 MPa. Areas in which yield limit (270 MPa) has been exceeded are marked with gray color.

**Figure 17:**
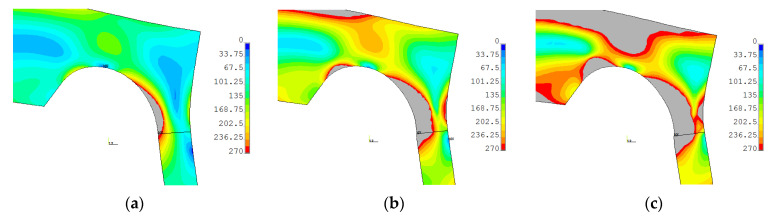
Equivalent von Mises stress in optimal SRG with mean dimensions, material 2P for: (**a**) cycle 6–22 MPa; (**b**) cycle 14–35 MPa; (**c**) cycle 26–41 MPa. Areas in which yield limit (270 MPa) has been exceeded are marked with gray color.

The development of the plastic zones for both non-optimal and optimal SRG investigated in the paper for operating pressure 22 MPa, water-proof test 35 MPa and the maximal 41 MPa are presented in Figure 16 and Figure 17. In these contour maps, the horizontal continuous black line represents the center of the weld joint of the flat end with the pipe. In the case of the optimal SRG, the stress concentration is located close to the weld joint and under operating pressure plastic stress occurs in the area of the weld joint and heat-affected zone (the Yield limit of the material is set to 270 MPa—see Table A2). This results in overlaps of the residual stresses and stresses caused by internal pressures and leads to higher deformations in the first measurement series (see results in Section 3.1.2). In the case of non-optimal SRG stress concentration is located at a greater distance from the weld joint and the influence of residual welding stresses is unnoticed in the measurements (Figure 13, Figure 14 and Figure 15). Despite this, the equivalent stresses and total equivalent strains as well as the plastic zones areas are significantly larger in non-optimal geometry. Moreover, it can be observed that for pressure 41 MPa plastic joint occurred in non-optimal SRG (Figure 16c), while in the optimal SRG there is still some reserve of structure load capability (Figure 17c).

**Figure 18:**
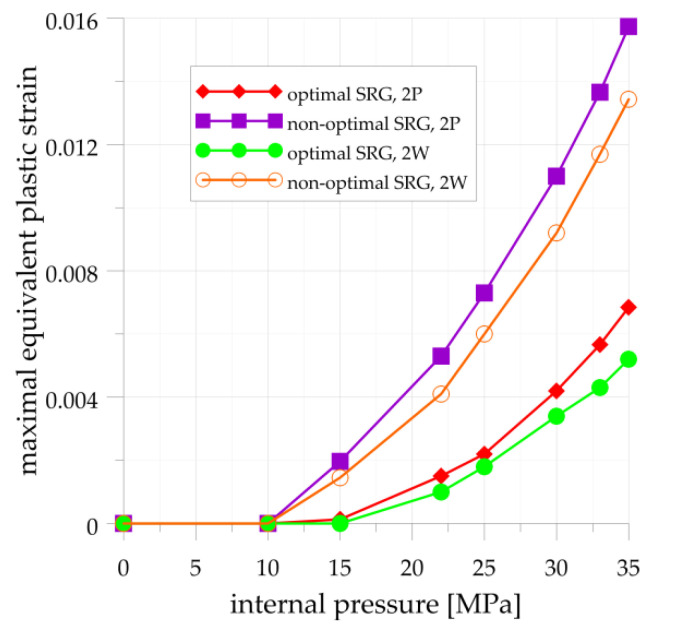
Maximal equivalent plastic strain in optimal and non-optimal SRG.

The relationship between the maximal equivalent plastic strain and internal pressure is plotted in Figure 18. The results are presented for both tested structures and calculated for the materials with the highest (2W) and the lowest (2P) Yield limits. It can be seen that the first plastic strains occur for loading lower than operating pressure 22 MPa. Regardless of the material, such plastic strains are greater and appear earlier in the non-optimal SRG than in the optimal SRG and in the cylindrical pipe. Moreover, the difference between the optimal and non-optimal shape of SRG may result in 2–3 times larger elastic-plastic strains in non-optimal geometry. In the examined examples, the total equivalent elastic-plastic strain is 2.6 and 2.2 times bigger in the non-optimal SRG for the operating pressure 22 MPa and 40 MPa, respectively.

### 3.2. Numerical Analyses of Different Flat Ends

All numerical calculation presented in this section are made with the following assumptions:

- The pure elastic material model is assumed;
- The operating pressure *p_c_* = 18.6335 MPa is calculated for nominal mechanical properties of the 16Mo3 steel (*R_eH_* = 270 MPa—Table A2);
- The nominal dimensions of the pipe are assumed as Ø406.4 × 20 mm.

#### 3.2.1. Bottom Type “a”

**Figure 19:**
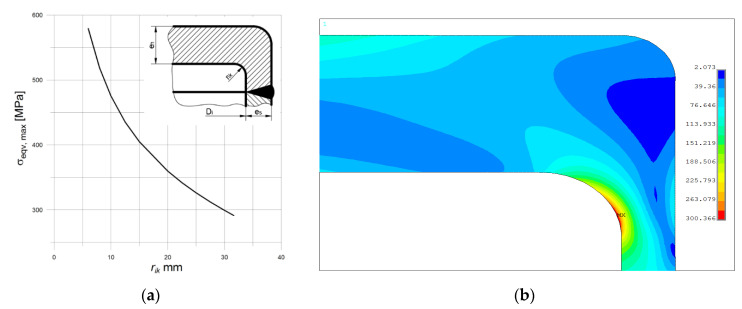
Bottom type “a”: (**a**) Maximum equivalent stress vs. inner radius; (**b**) distribution of equivalent stress in the stress concentration area, *r_ik_* = 30 mm.

Such kind of head/bottom type is the typical forged or machined one. Here (Figure 19), the calculated thickness of the endplate is equal to *e_h_* = 63.22 mm, the same value refers also to endplate types: “b”, “e”, “f”, and “h” (see Figure 1) as well.

According to the code recommendations [6], the inner radius *r_ik_* should be not less than 0.3 × *e_s_*, which means at least 6 mm in the analyzed case. Surprisingly, there is no limit for the upper value for *r_ik_* and the series of tests made for different values of this radius shows the strong influence of the inner radius on the maximum, observed equivalent stress. In this case, also rounded outer edge is introduced, but the influence of its value—*r_out_*—on the results is rather small and negligible. So that, for the lowest value for *r_ik_* = 6 mm and *r_out_* set to 20 mm, the maximum equivalent stress is equal to 583.38 MPa, while for *r_ik_* = 30 mm and *r_out_* as above maximum equivalent stress goes down to 300.5 MPa. In the code [6], no clear evidence is given on how to choose the *r_ik_* value, but choosing its minimal value evidently results in very high-stress concentration (see Figure 19a). For the last combination of dimensions, the illustration for equivalent stress distribution and stress concentration is shown below in Figure 19. Here, the minimum value of λ is equal to 1.67.

#### 3.2.2. Bottom Type “b”

This type is similar to that one described above, and the only difference is the presence of the sharp outer edge. Here, the minimum value for the *r_ik_* is set as previously, and its big influence on the maximum equivalent stress is observed. For the values of *r_ik_* chosen as previously the respective maximum stresses are as follow: σ_eqv,max_ = 579.2 MPa for *r_ik_* = 6 mm and σ_eqv,max_ = 299.5 MPa for *r_ik_* = 30 mm. The observed differences between the respective results for type “a” are very small so that both types can be regarded as equivalent (λ is equal to 1.66 for *r_ik_* = 30 mm).

#### 3.2.3. Bottom Type “c”, “d” and “g”

**Figure 20:**
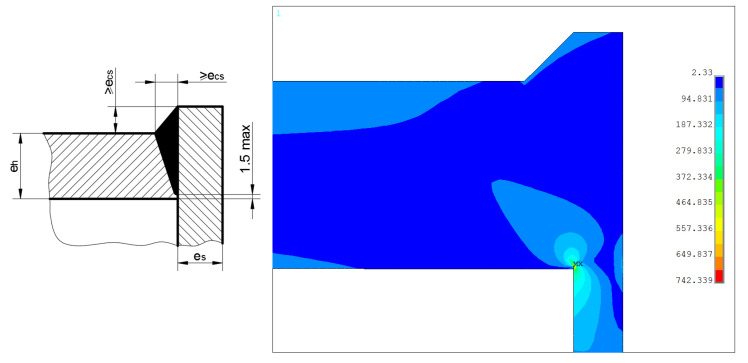
Equivalent stress distribution in the endplate “d”—endplate—tube junction.

These three types of the vessel ends are similar due to the presence of non-complete penetration weld, which is the common feature of them all. Here, the design type “d” is the subject of the numerical analysis. Due to the presence of the very sharp notch (at the root of the weld), the solution strongly depends on the element size, particularly around the notch area. For one of the studied approximations, the maximum numerically calculated equivalent stress exceeded the value of 740 MPa, which is completely unacceptable for designing purposes (see Figure 20). Here, λ is over 4.1. In this case, only the welds with complete penetration could be the remedy. A detailed and thorough study of the welds shapes is recommended here, which is beyond the scope of the presented paper.

#### 3.2.4. Bottom Type “e”

**Figure 21:**
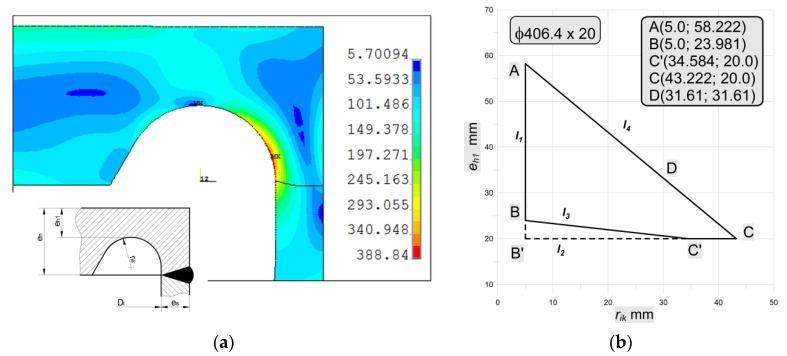
Bottom type “e”: (**a**) distribution of equivalent stresses in the groove for optimally chosen *r_ik_*; (**b**) Admissible area for head type “e”, for pipe with dimensions Ø406.4 × 20 mm.

This bottom type is tested in the experimental and numerical analyses in Section 3.1. In the case of the nominal dimensions and Yield limit *R_eH_* = 270 [MPa] slight changes in the optimal shape occurred (Figure 21b). The main differences in comparison with studies in Section 3.1 concern the flat-end thickness (*e_h_* = 63.22 mm in this case), operating pressure (*pc* = 18.6335 MPa) and the optimal value of SRG radius (*r_ik_* = 31.61 mm)—see point D in Figure 21b. Due to the same behavior as is presented in Section 3.1. only the equivalent von Mises stress for optimal SRG is presented in Figure 21a.

#### 3.2.5. Bottom Type “f”

**Figure 22:**
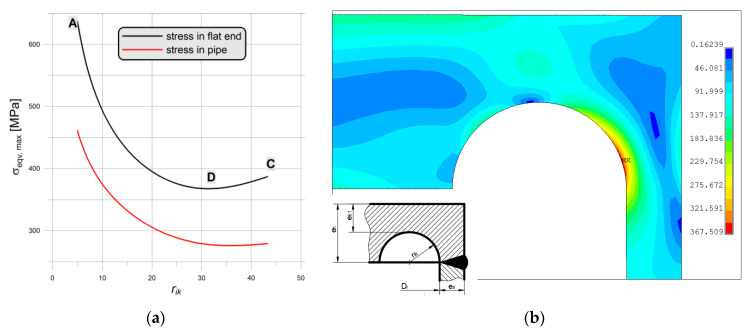
Bottom type “f”: (**a**) distribution of maximum equivalent stresses for pairs (*r_ik_*, *e_h_*_1_) placed along with line AC (see Figure 21b); (**b**) distribution of equivalent stresses in the groove for optimally chosen *r_ik_*.

Here, before the exemplification of the numerical results, a short important remark should be introduced. Following the results given in the paper [15] the influence of the inclination angle a on the numerical results becomes negligible when its value exceeds 60°, so that in the numerical analysis only grooves with the shape of the half-circle are investigated (Figure 22).

The polygonal area ABC’C (Figure 21b), which gives the admissible pairs of the groove radius *r_ik_* and the minimum thickness of the endplate in the grove *e_h_*_1_ is the same as for the solution of flat end type “e”. The performed numerical analysis made for pairs (*r_ik_*, *e_h_*_1_) over the whole area enabled to show the strong dependency between the resulting maximum equivalent stress and the value of the *r_ik_* and *e_h_*_1_. It is observed that the minimum stress concentration is observed for pairs located along with the line AC (see Figure 22a), in point C or its vicinity depending on the pipe diameter and the tube wall thickness. In the analyzed case the optimal value for *r_ik_* is depicted in point D—Figure 21b) and its value is equal to *r_ik_* = 31.61 mm. In Figure 22b, the equivalent stress distribution in the most strenuous area of the groove is shown, as it can be seen (Figure 22a) the maximum stress can vary from 367.6 MPa (for optimal point D, λ = 2.04) to 637.3 MPa (for non-optimal point A, λ = 3.54).

#### 3.2.6. Proposed Head with Elliptical SRG

**Figure 23:**
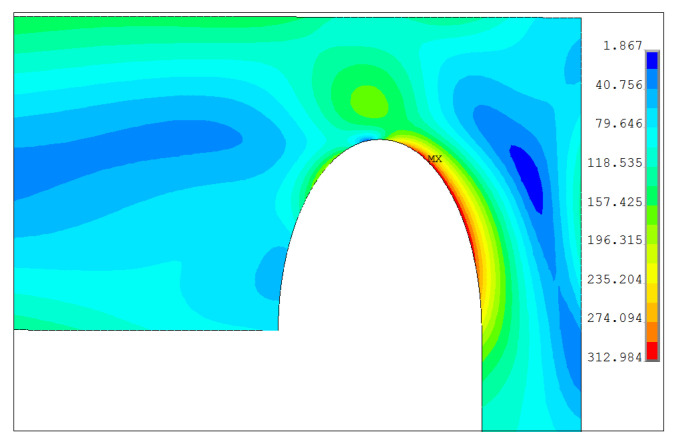
Distribution of equivalent stresses in the elliptical SRG for optimally chosen semi-axes.

It is common knowledge that in numerous cases the notches of the circular shape are not the best shapes [15,34]. Here, a certain reduction of the stress concentration is obtained when using the elliptical shape of the notch instead of the circular one. Here, the assumption that the maximum length of the vertical semi-axis would not cross the maximum value of *r_ik_* which is set for the circular groove. Below, in Figure 23 the equivalent stress distribution is shown for the elliptic groove. This solution is obtained for the mesh shown in Figure 9g. Here, the plot shows the results of the optimization when the maximum equivalent stress is the objective function. The optimization problem is solved with respect to the two design variables *b*—the length of the vertical semi-axis and the ratio between semi-axes κ = *b*/*a*. The maximum stress is equal to σ_eqv,max_ = 313 MPa (λ = 1.74) and is obtained for *b* = 38.63 mm and κ = 1.88. It is worth noticing that the value of *b* does not correspond to the optimal value of *r_ik_*. Here, the effectiveness of the stress reduction in comparison with the optimal circular groove is over 10 percent.

#### 3.2.7. Bottom Type “h”

**Figure 24:**
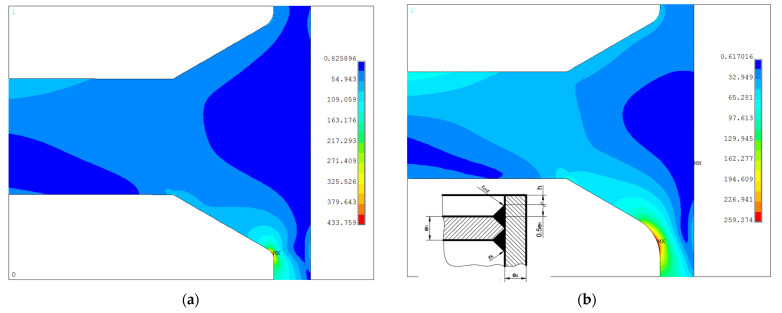
Bottom type “h”: (**a**) distribution of equivalent stresses in the groove for *r_ik_* = 6 mm; (**b**) distribution of equivalent stresses in the groove for *r_ik_* = 25 mm.

The last analyzed bottom type is this one shown in Figure 1h and Figure 24, here, for the pipe Ø406.4 × 20 mm the endplate thickness is the same as for the previously studied types “a”, “b”, “e”, and “f”. The code [6] gives also an additional remark concerning the minimum value of the radius *r_ik_*, which, as in the bottom case “a” and “b” should not be less than 0.3 × *e_s_*. Again, there is no upper limit for *r_ik_* value, which can result from the oversized inner weld (weld reinforcement). Additionally, the length of the protruding part of the pipe *h* is not specified in the code [6]. Here, the angle of the throat of the weld is set to 60°, which is the standard value, and then the size of the protruding part of the pipe is equal to 0.5 × *e_h_* + *h*. In the code [6] there is no limit for such value. In the series of the numerical test appeared that the maximum equivalent stress value strongly depended on two variables, the height *h* and the radius *r_ik_*. The outer radius *r_out_* had a negligible influence on the results if its value is set at minimum 0.3 × *e_s_*.

In order to illustrate this influence, the results for two combinations of *h* and *r_ik_* are cited below:

1. *h* = 50 mm, *r_ik_* = 6 mm, *r_out_* = 10 mm, σ_eqv,max_ = 433.8 MPa (λ = 2.44),
2. *h* = 50 mm, *r_ik_* = 25 mm, *r_out_* = 10 mm, σ_eqv,max_ = 259.3 MPa (λ = 1.44).

The comparison of the distributions of the equivalent stresses for both above-listed cases is shown in Figure 24.

Further increase of *h* and *r_ik_* leads to the reduction of the maximum equivalent stress, enough to say that for the combination *h* = 90 mm, *r_ik_* = 70 mm, *r_out_* = 10 mm, the maximum equivalent stress is reduced σ_eqv,max_ = 175.6 MPa (λ = 0.98). In this case, the stress maximum appears in the tube part—no longer notch effect in the junction between the tank and the flat bottom is observed. However, one must be aware of the fact that in this case, the endplate starts to behave like a baffle in the tank and not like the endplate. Additionally, the relatively high value of the inner radius seems to be unjustified from a designing point of view—highly oversized weld reinforcement.

#### 3.2.8. Numerical Studies of Vessel with Moderate Thickness

**Figure 25:**
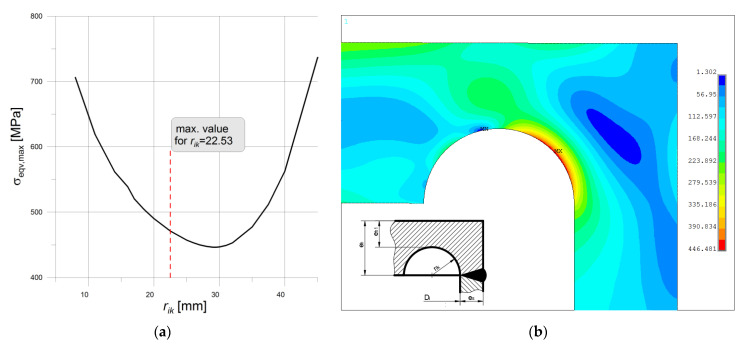
Bottom type “f”, pipe Ø406.4 × 40 mm: (**a**) maximum equivalent stress in function of *r_ik_*; (**b**) equivalent stress distribution for *r_ik_* = 29.25 mm.

It is mentioned previously that the flat ends are particularly useful in the case of the tank with higher pipe wall thickness. The comparative analysis is performed for the pipe Ø406.4 × 40 mm with the two types of ends, namely “f” and “h”. The motivation for this is almost the same endplate thickness as for the pipe with *e_s_* = 20 mm. Here, the endplate thickness is equal to *e_h_* = 62.53 mm (see Figure 2b), while the calculated pressure for the tank is set to *p_c_* = 39.301 MPa. In the case of the endplate type “f” the admissible values for *r_ik_* calculated on the base of the set formulas given in EN 12952-3 gave rather a small area of choice with values ranging from 8.0 mm at minimum to 22.53 mm at maximum. This strongly limits the area of search for the optimal value for *r_ik_* but again the optimal solution fulfilled the condition: that *r_ik_* + *e_h_*_1_ = *e_h_*, which meant that the optimum is located on the line AC (see Figure 21). For the admissible range of *r_ik_*, the minimum of the stress concentration is observed for the maximum admissible value of *r_ik_* = 22.53 mm. However, the question arose whether this value provided the minimum value for the maximum stress at all. To study this the values for *r_ik_* greater than the maximum admissible are introduced and numerically analyzed. The performed study revealed that in this case the maximum is far beyond the admissible range and is reached for the higher value of *r_ik_*, namely 29.25 mm. This is illustrated in Figure 25 below, where the dependency between the maximum equivalent stress and the *r_ik_* is shown (Figure 25a), and the respective equivalent stress distribution in the vessel for *r_ik_* = 29.25 mm is shown in Figure 25b.

**Figure 26:**
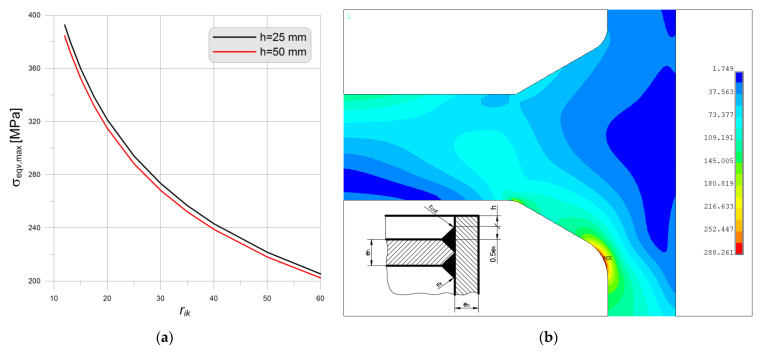
Bottom type “h”, pipe Ø406.4 × 40 mm: (**a**) maximum equivalent stress in function of *r_ik_* (**b**) equivalent stress distribution for *h* = 50 mm and *r_ik_* = 25 mm.

A similar analysis for the flat end type “h” is performed. The pressure and the endplate thickness are the same as above, for the type “f”. In this study, the dependency between the maximum equivalent stress in the notch and the radius *r_ik_* is searched. It is observed that the maximum equivalent stress as previously strongly depended on the *r_ik_*, while the influence of the height *h* (see Figure 26a) is rather small. The stress distribution shown in Figure 26b indicates that the increase of the pipe wall and the accompanying to this dimensions pressure *p_c_* = 39.301 MPa worsen the situation, parameter λ is equal 1.60 (for *r_ik_* = 25 mm), which is worse than for the pipe with the wall thickness 20 mm. However, it still much better than for the respective solution observed for the head type “f”.

#### 3.2.9. Summary of Numerical Study

The final summary of the numerical analyses presented in Section 3.2 is given in Table 3. Such comparison and the selection of the optimal flat end geometry is based on the maximal equivalent von Mises stress. It can be seen that definitely the worst solution is obtained for the flat end type “d”, where very high-stress concentration is observed at the root of the weld. The same situation appeared for endplates type “c” and “g”, for which the numerical results are not presented. The best result is obtained for the head type “h” but for the strongly exaggerated dimensions of the protruding part of the tube (*h*) and the for the big T-weld reinforcement. If the values of *r_ik_* assumed for the heads type “a”, “b”, “h”, remained in the reasonable ranges then the λ parameter varied from 1.44 (in the best case) to 3.24 (in the worst case). The endplates type “e” and its modification in the “f” form were formerly studied in papers [9,15,16] with the elastic-plastic properties of the materials applied. Here, the study performed with only elastic properties confirmed the existence of the optimal point, in which the minimum stress concentration is observed.

For various combinations of admissible dimensions, the common feature is observed—the location of the optimum along with the line AC (see Figure 21b) where *r_ik_* + *e_h_*_1_ = *e_h_*. With the increase of the wall thickness, the point D tends toward vertex C and finally overlaps with point C. In the case of the endplate with SRG certain improvement in reduction of stress concentration is observed when the shape is switched from circular one to the elliptical one. For the pipe Ø406.4 × 20 mm in the performed calculations about 15% stress reduction is registered, and finally λ dropped down to 1.74. The main drawback of the application of various SRGs is the necessity of the application of optimization procedures to get the optimal solution. The obtained values of λ for endplates with SRGs are not much worse than these minimal obtained for “a”, “b” or “g”.

Summarizing, taking into account the only criterion of the lowest equivalent von Mises stress there is no unequivocal answer which type of endplate is the best one and recommended for use. This is due to the possibility of changing the endplate geometry (i.e., theoretically unlimited value of the radius *r_ik_* or endplate thickness *e_h_*) to achieve the demanded load capability. However, the increase of the key geometric parameters of the bottom leads to caricatured and impractical solutions. Because of this, an additional parameter taking into account the minimal endplate thickness required for manufacturing *e_B,MIN_* and the maximal equivalent von Mises stress is introduced:

(4) $$\xi =\frac{e_{B,MIN}\times \sigma_{\mathrm{eqv},\max}}{1000\;\left[\mathrm{Nmm}\right]}.$$

In this comparison, it is assumed that the bottom is made from the flat slab by machining and/or welding. In such a situation, for bottoms with geometry “a” and “b” the minimal thickness is at least the sum of *e_h_* and *r_ik_*, for bottoms with geometry “e”, “f” and with elliptical SRG the minimal thickness of the slab is exactly equal to the *e_h_*, and in the case “h” *e_B,MIN_* is assumed as the required increased length of the pipe and calculated as *e_B,MIN_* = *e_h_* + *h* + 0.5 *e_h_*. All results are given in Table 3.

Based on the comparison of the parameter ξ (smaller ξ means better solution), the optimal geometry is the geometry “f” with elliptical SRG (ξ = 1.98), which is not included in the Standards [6]. Other solutions which show relatively low parameter ξ are the flat-ends with circular cutouts type “e” (ξ = 2.46) and “f” (ξ = 2.32). Anyhow, the common conclusion is that in the case of the flat endplate use the pressure reduction, calculated for the pipe and its wall thickness, is demanded to provide safe structure operation.

## 4. Discussion

The presented in the paper results and the comparisons of the experimental and numerical studies for the non-annealed and annealed vessel allow to conclude that the manufacturing procedure and selection of the flat end shape have a significant influence on the safe operation of the pressure vessel. In the case of the flat end with the optimal shape of the stress relief groove, the residual welding stresses significantly decrease the strength of the structure. Because of this, annealing of the vessel after welding is necessary or even obligatory. On the other hand, the optimal geometry is less sensitive to a change of dimensions due to its tolerances and reveals significantly smaller equivalent stresses than in the non-optimal one. In the second investigated case of the non-optimal SRG, no differences are observed before and after annealing. It seems that the residual welding stresses did not significantly influence the elastic-plastic behavior of the non-optimal shape. However, such non-optimal geometry is very vulnerable to change of dimensions due to its tolerances. It can be explained by the small fillet radius of the stress relief groove. Regardless of the SRG shape, the flat end is a part in which zones of plastic stress appear first (in comparison with the cylindrical pipe without any holes). For this reason, it is reasonable to optimize the geometry of the flat end and use material with a higher Yield limit for the vessel heads.

**Figure 27:**
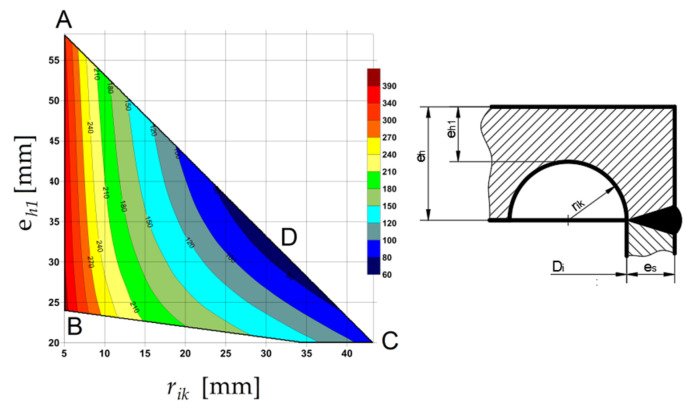
Equivalent strain distribution in the admissible area for (*r_ik_*, *e_h_*_1_) pairs.

The optimization of the SRG presented in the Reference [15] for the endplate “e”, as well as the numerical studies presented in the paper revealed that the choice of the geometrical parameters of SRG (see Figure 21) given in the Standards [6] is not sufficiently defined. The admissible range for *r_ik_* and *e_h_*_1_ (area with the polygonal shape) allows for the selection of the radius *r_ik_* in a wide range, and no suggestion is given in code how to optimally choose its value. To illustrate the importance of this choice the elastic-plastic analysis of the tank with the head type “f” is performed. Here, the tension curve with the linear hardening is introduced, and the hardening modulus *E_t_* is assumed on the base of the *E*, *R_e_*_H_, *R_m_* and *A*_5_ values. The obtained numerically distribution of the equivalent plastic strains (Figure 27), for all admissible pairs of *r_ik_* and *e_h_*_1_, clearly shows the big discrepancy of results between the optimal and non-optimal choice of the SRG dimensions. It is observed that the optimal point providing the minimum value for equivalent stress or strain (D) lies on the line AC and its location close to the corner C and the non-optimal point lies on line AB (Figure 27). This general tendency is confirmed for different combinations of the pipe wall thickness, and with the increase of the pipe wall, the point D moves toward the point C. It means, that the optimum for higher values if the pipe thickness can be assigned to the maximum allowable value of *r_ik_*. The results presented in Figure 27 were calculated for material with linear hardening. It is also obvious that the change of the material properties affects the solution (mainly the Yield limit).

The all numerical results confirm that in the case of vessels with the flat endplates the operating pressure calculated on the base of the strength of the pipe cannot be longer applied in operation. In this situation, a series of numerical calculations should be made to get the final value of the maximum pressure. Because of this, the optimization of the flat ends with stress relief grooves is an important design issue.

The study of the obtained numerical results shows the strong influence of *r_ik_* value on the result is observed for three geometries of the endplates, namely “a”, “b” and “h”. For large *r_ik_* it is possible to get the smallest equivalent stress. On the other hand, the application of a relatively high value for *r_ik_* in the case of head type “h” is accompanied by the big weld reinforcement, which in general is not recommended in practical applications and demands the performing the cost generating the post-welding heat treatment. The endplates “c”, “d” and “g” are not recommended for use in the form proposed in EN 12952-3 [6]. In this case, only the use of a full-penetration weld joining head with tubes may be the possible improvement and may lead to the reduction of the stress concentration.

## 5. Conclusions

The numerical and experimental studies of the standardized flat ends are carried in the paper. On this basis, the optimization and comparison of the allowable flat end geometries are performed. Finally, the following conclusions can be drawn:

- The flat-ends with SRG are the most optimal. The proposed elliptical shapes for SRG appeared to be even more effective than the circular one,
- The performed numerical tests exemplified the high differences in results for pairs (*r_ik_*, *e_h_*_1_) taken from the admissible area for both circular and elliptical SRG studied,
- The comparison of experimental tests with the numerical study confirms the proper selection of the optimal shape of the SRG for the bottom type “e”,
- For the pipe with a higher value of the wall thickness (i.e., 40 mm) and with the SRG of circular shape, the optimal configuration is reached for *r_ik_* beyond the admissible range,
- The residual welding stresses have a significant influence on the stress and strain level. Such effect is stronger in optimal SRG due to the vicinity of the stress concentration area to the weld zone,
- The slight differences in geometry (due to the tolerances) may lead to a significant increase of stresses and deformations particularly in the case of non-optimal SRG.

## Figures and Tables

**Table 1:** Measured and nominal dimensions of tested flat ends with stress relief grooves.

Geometry	Part	*e_s_* (mm)	*h* (mm)	*r_ik_* (mm)	*α* (deg)	*D_i_* (mm)	*D_o_* (mm)	*e_h_*_1_ (mm)	*e_h_* (mm)	Remarks
1 (opt.)	Head	20.6	0	30	60	364.8	406	31.3	61.3	Measured in situ (mean)
1 (opt.)	Pipe	22.0	-	-	-	359.9	403.9	-	-	Measured in situ (mean)
2 (non-opt.)	Head	20.9	26.5	5	60	364.3	406	29.65	61.15	Measured in situ (mean)
2 (non-opt.)	Pipe	21.7	-	-	-	359.9	403.3	-	-	Measured in situ (mean)
1 (opt.)	Head	20.0	0	29.8	60	366.4	406.4	31.2	-	Nominal dimensions [17]
2 (non-opt.)	Head	20.0	26.76	5	60	366.4	406.4	29.24	-	Nominal dimensions [17]

**Table 2:** Position of strain gauge chains. Distance *SGy* determines center of first strain gauge from end of vessel.

Strain Gauge Chain	T1,1	T1,2	T1,3	T1,4	T2,1	T2,2	T2,3	T2,4
*SGy* (mm)	36	36	23	23	39	39	23	23

**Table 3:** Summary of numerical results for investigated flat ends with different geometries, pure elastic model, *p_c_* = 18.6335 MPa.

Geometry	Characteristic Parameters	*e_h_* (mm)	σ_eqv,max_ (MPa)	λ	*e_B,MIN_* (mm)	ξ	Remarks
“a”	*r_ik_* = 6 mm, *r_out_* = 20 mm	63.22	583.4	3.24	69.22	4.04	Solution for minimal *r_ik_*
	*r_ik_* = 30 mm, *r_out_* = 20 mm	63.22	300.4	1.67	93.22	2.80	There is no upper limit for *r_ik_*
“b”	*r_ik_* = 6 mm, *r_out_* = 20 mm	63.22	579.2	3.22	69.22	4.01	Solution for minimal *r_ik_*
	*r_ik_* = 30 mm, *r_out_* = 20 mm	63.22	299.5	1.66	93.22	2.79	There is no upper limit for *r_ik_*
“d”	*e_cs_* = 20 mm	76.30	742.3	4.1	76.30	5.66	Stress concentration at the root of the weld
“e”	*r_ik_* = 5 mm	63.22	593.3	3.30	63.22	3.75	Optimal
“e”	*r_ik_* = 31.61 mm	63.22	388.8	2.16	63.22	2.46	Non-optimal
“f”	*r_ik_* = 5 mm	63.22	637.3	3.54	63.22	4.03	Point A (Figure 21 and Figure 22)
	*r_ik_* = 43.22 mm	63.22	386.8	2.15	63.22	2.45	Point C (Figure 21 and Figure 22)
	*r_ik_* = 31.61 mm	63.22	367.6	2.04	63.22	2.32	Optimal—point D (Figure 22)
elliptical SRG	*b* = 38.63 mm, κ = 1.88	63.22	313.0	1.74	63.22	1.98	Optimal; *a*, *b*—horizontal and vertical semi-axes, κ = *b*/*a*
“h”	*r_ik_* = 6 mm, *r_out_* = 10 mm, *h* = 50 mm	63.22	433.8	2.44	144.83	6.28	Solution for minimal *r_ik_*, *r_out_* and *h* are arbitrarily chosen
	*r_ik_* = 25 mm, *r_out_* = 10 mm, *h* = 50 mm	63.22	259.3	1.44	144.83	3.76	There is no upper limit for *r_ik_*, *r_out_* and *h* are arbitrarily chosen
	*r_ik_* = 70 mm, *r_out_* = 10 mm, *h* = 90 mm	63.22	175.6	0.98	184.83	3.25	There is no upper limit for *r_ik_*, no practical case due to large *h* value

**Figure A5:**
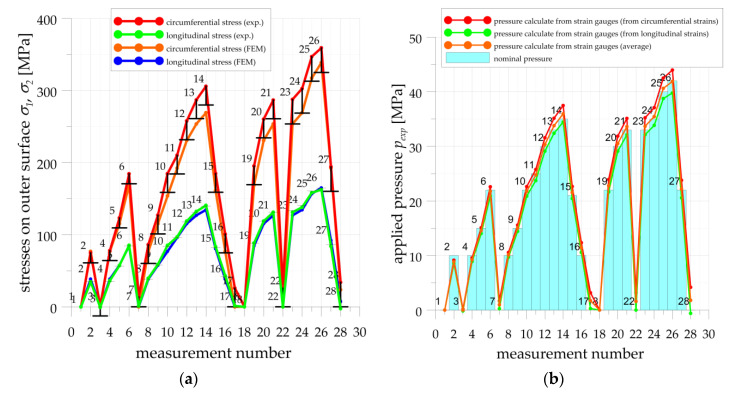
Results obtained from rosette T2.5: (**a**) experimental and numerical principal stresses on outer surface of cylindrical pipe; (**b**) calculated internal pressure from strain measurements.

**Figure A6:**
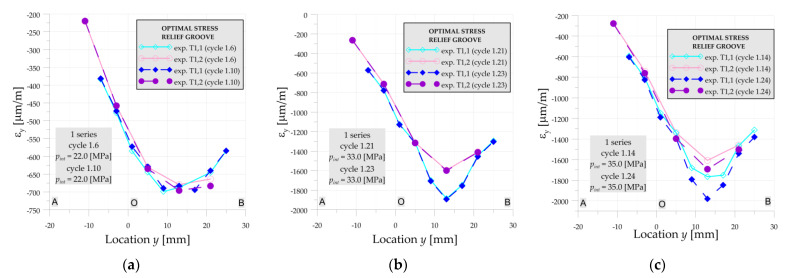
Repeatability of strain gauge measurements, optimal SRG, 1st series: (**a**) cycles 6 and 10; (**b**) cycles 21 and 23; (**c**) cycles 14 and 24.

**Figure A7:**
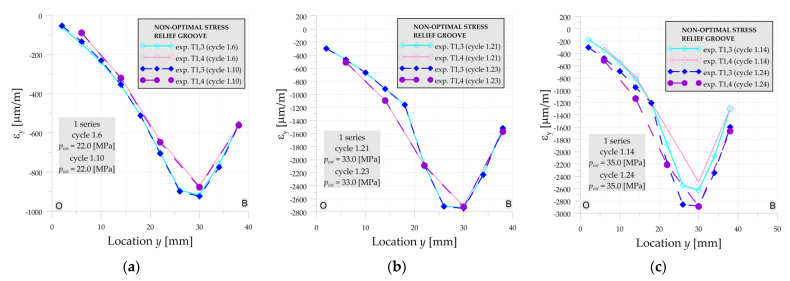
Repeatability of strain gauge measurements, non-optimal SRG, 1st series: (**a**) cycles 6 and 10; (**b**) cycles 21 and 23; (**c**) cycles 14 and 24.

**Figure A8:**
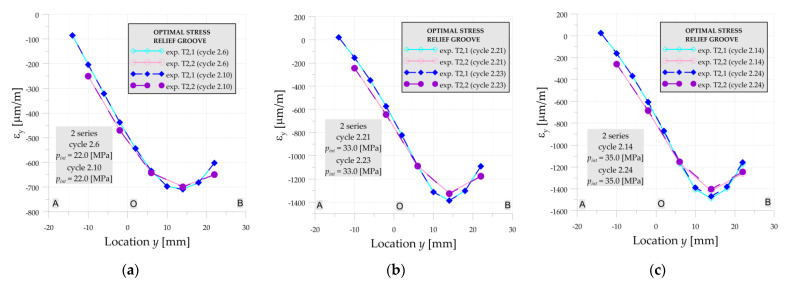
Repeatability of strain gauge measurements, optimal SRG, 2nd series: (**a**) cycles 6 and 10; (**b**) cycles 21 and 23; (**c**) cycles 14 and 24.

**Figure A9:**
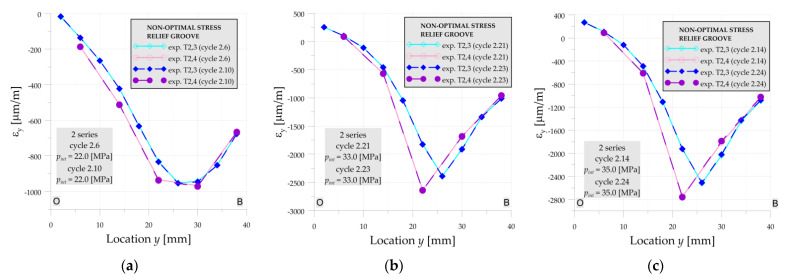
Repeatability of strain gauge measurements, non-optimal SRG, 2nd series: (**a**) cycles 6 and 10; (**b**) cycles 21 and 23; (**c**) cycles 14 and 24.

## Data Availability

Not applicable.

## Appendix A. Experimental Tensile Tests

The determined chemical composition of the used material in manufacturing the tested vessel as well as requirements for such material specified in the Standards are given in Table A1. Due to the chemical composition, 16Mo3 has good tensile strength, yield limit, fracture toughness and welding properties, as well as, high corrosion, heat and wear resistance.

**Table A1:** Chemical composition of experimentally tested chrome-molybdenum 16Mo3 alloy steel (in % by weight).

Material		C	Mn	Si	P	S	Cr	Ni	Cu	Mo	Al	N	Sn
Tested material 16Mo3 (set 1)		0.15	0.57	0.24	0.014	0.002	0.09	0.08	0.20	0.27	0.028	0.009	0.012
16Mo3 (standards [37])	Min	0.12	0.40	−	−	−	−	−	−	0.25			
	max	0.20	0.90	0.35	0.025	0.010	0.30	0.30	0.30	0.35		0.012	

The experimental tensile tests were performed for samples made of 16Mo3 material used for manufacturing the flat end and the cylindrical pipe. The three sets of the samples were cut from different parts of the investigated pressure vessel. The first set of the samples (1a and 1b in Table A2) with diameter Ø15 mm, were cut from the plate used for the fabrication of the vessel head. The second set of the samples (2a and 2b in Table A2) with diameter Ø10 mm, were cut from the pipe used for the fabrication of the cylindrical part of the pressure vessel. In both above cases, the 7 samples were cut for a particular set in each of the longitudinal and transverse directions to the vessel axis. The third set of the 7 samples (set 3 in Table A2) with a diameter Ø10 mm, were cut in the transverse direction from the butt joint of the pipes with a diameter Ø406 × 20 mm. The connections were made by arc welding and the welding positions were at a flat position. The experimental tests were carried in accordance with the EN 10002-1 Standards [38] at room temperature 20 °C. The mechanical properties of 16Mo3 + QT after heat treatment (set 4) and the minimal mechanical properties of 16Mo3 + N (given in Standards [37]—set 5) are also reported in Table A2.

The results of the experimental tests are summarized in Table A2. The average values and the standard deviations of the upper Yield limit *R_eH_*, tensile strength *R_m_*, elongation after fracture *A*_5_ and reduction of area *Z* are calculated by the use of the 7 samples for a particular set.

**Table A2:** Mechanical properties of chrome-molybdenum 16Mo3 alloy steel.

Set No.	Material ^1^, Location of Sample, Direction of Sample		*R_eH_* MPa	*R_m_* MPa	*A*_5_ %	*Z* %
1a	16Mo3 + N, flat end, longitudinal ^2^		299.3 (8.59)	441.6 (8.77)	36.8 (1.45)	74.9 (0.49)
1b	16Mo3 + N, flat end, transversal ^2^		284.2 (6.55)	449.1 (3.95)	33.6 (1.63)	72.3 (0.82)
2a	16Mo3 + N, pipe, longitudinal ^2^		309.1 (1.21)	515.9 (1.68)	29.0 (1.41)	60.8 (1.61)
2b	16Mo3 + N, pipe, transversal ^2^		317.3 (4.19)	518.4 (0.79)	28.0 (0.82)	61.2 (1.72)
3	16Mo3 + N, welded joint, transversal ^2^		384.7 (3.59)	537.3 (2.21)	18.4 (1.13)	60.2 (2.10)
4	16Mo3 + QT		382	452	35.0	72.9
5	16Mo3 + N (standards [37]) ^3^	Min	270	440	22	
		max		590		

^1^ +N = normalized, +QT = quenched and tempered (Q—890/960 °C in oil, T—620/700 °C in water), ^2^ average values and standard deviation (in brackets) for 7 samples, ^3^ data for samples with thickness in the range of 16–40 mm.

The approximated engineering σ-ε experimental curves of the material used for manufacturing the flat ends (16Mo3 + N) are presented in Figure A1 and Figure A2.

These obtained σ-ε curves have the typical behavior of low-carbon steels. After reaching the upper yield strength, the stress suddenly drops and fluctuates about some constant value of stress and after that increases with strain. Slight anisotropy of the mechanical properties of flat end material is observed. Samples cut in the longitudinal direction (Figure A1) had a higher average upper Yield limit (about 5%), elongation after fracture (9%), reduction of area (4%) but lower tensile strength (2%) than samples cut in transversal direction (Figure A2). The minimal and the maximal upper Yield limit for the longitudinal samples are equal to 287.3 MPa (sample 6W) and 305.5 MPa (sample 2W), respectively and for the transversal samples are equal to 274.1 MPa (sample 2P) and 294.4 MPa (sample 3P), respectively.

Slight anisotropy, but much smaller than for flat end (set 1 in Table A2), is also observed for samples cut from the pipe (set 2 in Table A2). However, in this case, a higher upper Yield limit (2.5%) and tensile strength (0.5%) show samples cut in the transversal direction. The lowest Yield limit observed for samples cut from the pipe is equal to 308 MPa.

The samples cut from the pipe revealed higher strength than samples cut from the flat end. Comparing the worst average values for these parts of the pressure vessel, the yield limit of the samples cut from the pipe is higher by 9% and tensile strength by 17%. Such differences are caused by different manufacturing of both parts. The flat end was made of smith forging, which was delivered in quench-tempered condition but the pipe was supplied in the annealed condition. Such difference in material properties has significant meaning in the design process of the pressure vessels, because the highest stress concentration may occur in the stress relief groove of the flat end or in the surroundings of the place where the flat end is welded to the cylindrical part of the vessel.

The mechanical properties are also experimentally determined for the butt welded joint (set 3 in Table A2). Due to the welding process, a significant increase in the Yield limit and tensile strength is observed. The value of the Yield limit is comparable with the Yield limit of the non-welded and heat-treated samples made of 16Mo3 (set 4 in Table A2). In the comparison of such results (set 3) with the smallest mechanical properties of the material used for the flat end of pressure vessel (set 1) it can be seen that the Yield limit of the welded joint is increased by 35% and tensile strength by 22%. However, the increase of these parameters caused a significant reduction of the elongation after fracture (it is almost two times smaller). The lowest Yield limit for samples cut from the welded joint is equal to 380 MPa.

The determined mechanical properties and material behavior (σ-ε curves) of particular sets of the samples are used in the numerical FEM analyses for comparison and verification of the numerical and experimental tests of the pressure vessels.

## Appendix B. Measurements of Cylindrical Pipe

The nominal diameter and thickness of the cylindrical pipe of the vessel are Ø406.4 × 20 mm respectively. However, the real dimensions are limited by the tolerances of the pipe. Such tolerances are given in the Standard [36]. In the case of the pipe, the admissible tolerance on the outside diameter for class D1 is equal to ±1.5% (with minimum ±0.75 mm) and the tolerance on the thickness for class T1 is equal to ±15% (with minimum ±0.6 mm). In the investigated vessel it means deviations ±6.09 mm on the outside diameter of the vessel and ±3.3 mm on the thickness of the cylindrical part. The first numerical analyses performed for nominal dimensions revealed significant differences between numerical and experimental results [17]. Because of this, detailed measurements of the cylindrical pipe are carried. The outside diameter is measured in the 11 cross-sections with every 120 mm. The cross-section no.1 defines the geometry of the welded joint with the optimal stress relief groove, while the cross-section no.11 for the worst (non-optimal) geometry of the stress relief groove. The diameter is measured in the four planes every 45°: plane 1—0°–180°, plane 2—45°–225°, plane 3—90°–270°, plane 4—135°–315°. The details and results of the measurements are summarized in Figure A3. In all measured cases, the outside diameter had a negative deviation in respect to the nominal diameter Ø406.4 mm. The minimal diameters in the cross-sections no.1 and no.11 are Ø402.8 mm and Ø402.2 mm, respectively. The mean outside diameter from all measurements is equal to Ø403.4 mm.

The thickness of the cylindrical part of the vessel is measured in the same 11 cross-sections such as the outside diameter. The measurements are taken every 10 degrees, where θ = 0° means the top of the vessel and θ = 180° means the bottom of the vessel. The details and results of the pipe thickness measurements are given in Figure A4. The real thickness is larger than nominal (20.0 mm) in all measured points. The mean thickness of the cylindrical part calculated from all measurements is equal to 21.8 mm. The mean thicknesses in cross-sections no.1 and no.11 are equal to 22.0 mm and 21.7 mm, respectively, however, the measured deviations between the maximal and the minimal value of the thickness are significantly different (1.3 mm for cross-section no.1 whilst 2.2 mm for cross-section no.11). Moreover, in the cross-section no.11 (welded joint with non-optimal stress relief groove) the minimal thickness has reached the lowest value of all measurements taken (20.6 mm)—see Table A3 and Figure A4.

**Table A3:** Measured thickness of cylindrical pipe of pressure vessel—minimal, maximal and mean value of thickness in particular cross-sections.

Cross-Section Number	1	2	3	4	5	6	7	8	9	10	11
Min thickness (mm)	21.5	21.6	22.4	21.2	21.1	20.9	20.9	20.9	20.9	20.8	20.6
Max thickness (mm)	22.8	22.7	22.9	22.8	22.9	23.0	23.1	22.9	22.6	22.6	22.8
Mean thickness (mm)	22.0	22.0	22.0	21.9	21.9	21.8	21.8	21.8	21.7	21.6	21.7

## Appendix C. Measurements of Internal Pressure

In the 2nd measurements series, the internal pressure in the pressure vessel is calculated from the strains measured by the strain gauge 0°/60°/120° rosette T2.5 (Figure 3a).

In the elastic range the principal circumferential σ_1_ and longitudinal σ_2_ stresses are calculated as follows [39]:

(A1) $$\sigma_{1}=\frac{E}{1-\nu }\frac{\varepsilon_{a}+\varepsilon_{b}+\varepsilon_{c}}{3}+\frac{E}{1-\nu }\sqrt{{\left(\frac{2\varepsilon_{a}-\varepsilon_{b}-\varepsilon_{c}}{3}\right)}^{2}+\frac{{\left(\varepsilon_{b}-\varepsilon_{c}\right)}^{2}}{3}\;},$$

(A2) $$\sigma_{2}=\frac{E}{1-\nu }\frac{\varepsilon_{a}+\varepsilon_{b}+\varepsilon_{c}}{3}-\frac{E}{1-\nu }\sqrt{{\left(\frac{2\varepsilon_{a}-\varepsilon_{b}-\varepsilon_{c}}{3}\right)}^{2}+\frac{{\left(\varepsilon_{b}-\varepsilon_{c}\right)}^{2}}{3}\;}.$$

The principal stresses measured by the strain gauge rosette are presented in Figure A5a. The experimental results are compared with the results of numerical analyses. In the FEM model the pipe thickness (22.0 mm—angle θ = 90° in Figure A3), the outer diameter (Ø402.9 mm—plane number 3 in Figure A4) and the nominal pressures (Figure 7) are assumed based on the measurements of the cylindrical pipe geometry in cross-section 6 and location of the strain gauge rosette T2,5. A very good agreement of the experimental and numerical longitudinal stresses is achieved. On the other hand, the experimental circumferential stresses are higher than numerical results. It should be noted, that the first plastic deformations on the internal surface of the pipe occur in FE solution for the internal pressure equal to 40 MPa. Moreover, for both pressures 40 MPa and the maximal 41 MPa there were no plastic strains on the external surface of the pipe. However, the values for pressures in cycles 25–28 should be considered carefully because of the accuracy of the tolerances of the manufacturing of the wall thickness. This effect can be treated as some measurement error caused by the mechanical hysteresis of a strain gauge set and possible influence of the plastic deformations (see Table A4). This measurement error is determined on the basis of the stress values for the unloaded vessel (cycles 3, 7, 17, 22 and 28) and presented by error bars (black lines) in Figure A5a. Including error bars, the obtained experimental results are in good agreement with numerical ones and confirm the assumed pressure cycles (Figure 7). Finally, the pressures in particular cycles are determined from the principal stresses and compared with nominal pressures in Figure A5b. In the case of thin-walled pressure vessels the following formulations between internal pressure (*p_exp_*), pipe diameter (*D*), pipe wall-thickness (*e_s_*), and principal stresses (circumferential σ_1_ and axial σ_2_) are valid [7]:

(A3) $$\sigma_{1}=\frac{p_{exp}\cdot D}{2e_{s}},\;\;\;\sigma_{2}=\frac{p_{exp}\cdot D}{4e_{s}}.$$

In the investigated case, the internal pressure was calculated as the average value from both calculated principal stresses (Equations (A1) and (A2)) with the given below formula:

(A4) $$p_{exp}=0.5\left[\frac{2\sigma_{1}}{D}e_{s}+\frac{4\sigma_{2}}{D}e_{s}\right].\;$$

It can be seen, that in some cases of cycles with higher pressure (above 22 MPa) the applied average value of pressures are slightly higher than the nominal ones. Such differences are probably caused by the inertia of the pump, however, such good agreement of experimental and numerical stresses, as well as, calculated pressured from experimental stresses and nominal pressure allows to conclude that the experimental tests are carried out correctly.

**Table A4:** Internal pressures calculated from rosette T2.5 for measurements with unloaded vessel.

Measurement Number	1	3	7	17	18	22	28
*p_int_circ_* [MPa]	0	0.14	1.67	3.14	0	3.16	4.11
*p_int_long_* [MPa]	0	−0.14	0.27	0.29	0	0	−0.59
*p_int_avg_* [MPa]	0	0	0.97	1.72	0	1.56	1.76
Applied pressure [MPa]	0	0	0	0	0	0	0

## Appendix D. Verification of Experimental Tests

The accuracy and repeatability of the strain gauge measurement are also assessed based on the comparison of the experimental results for selected cycles for both optimal and non-optimal geometries. As it can be seen in Figure A5, the following pairs of the cycles are suitable for such comparison: 2–4, 6–10, 21–23 and 14–24. All numerical and experimental results are presented on the path related to the locations of the strain gauges (see Figure 3a and Figure 5b and Table 2). The location *y* is measured with respect to the center of the weld joint between the flat end and the cylindrical pipe. It is assumed that point “O” (*y* = 0) is the weld center, part “A-O” (*y* < 0) means strains in the pipe and part “O-B” (*y* > 0) means strains in the flat end.

In the case of the 1st series, the measurements were made in two sequences 1–17 and 18–28. The first cycles 2–4 (*p_int_* = 10 MPa) were performed to check the measurement system. The results obtained for cycles 2 and 4 have a similar manner to the results obtained for cycles 6 and 10 (*p_int_* = 22 MPa) and because of this are not presented. The accuracy and repeatability of the measurement system can be evaluated for results obtained for cycles 6 and 10 in Figure A6a and Figure A7a. In all cases (cycles 1–17) the acceptable high accuracy of measurements is achieved for both optimal and non-optimal SRG.

Due to the break after cycle number 17, the second sequence of the 1st series was started with zeroed strain gauge state (cycle 18). Because of this, the final state (cycle 17) was added to the results of all measurements of the second sequence of the 1st series (cycles 18–28). Such a break in the research program (17/18 cycles) was caused by the issues related to the evaluation of the experimental results (1–17 cycles) and its comparison with the numerical results and set up of the further research program and limitations. Such preliminary evaluation and verification of the results with the use of the FEM allow for the application of the higher internal pressure in the range up to 41 MPa. The high repeatability is also achieved for high pressures (*p_int_* = 33 MPa—cycles 21 and 23—Figure A6b and Figure A7b, however, slight differences occurred between results obtained for both sequences (*p_int_* = 35 MPa—cycles 14 and 24—Figure A6c and Figure A7c).

After 1st series, the pressure vessel has been annealed in order to eliminate residual stresses caused by the welding process and loading subjected in the 1st measurement series. The location of the new strain gauge chains is given in Table 2. All measurements in the 2nd series were made without any breaks and with the same loading cycles as it was applied in the 1st series.

The comparisons of the repeated cycles for both optimal and non-optimal SRG are presented in Figure A8 and Figure A9. As it can be observed high repeatability in 2nd series, as well as better than in the 1st series, is achieved for both SRG and including low and high pressures.
